# Cytosolic dopamine determines hypersensitivity to blunt force trauma

**DOI:** 10.1016/j.isci.2024.110094

**Published:** 2024-05-23

**Authors:** Kielen R. Zuurbier, Rene Solano Fonseca, Sonja L.B. Arneaud, Lexus Tatge, Gupse Otuzoglu, Jordan M. Wall, Peter M. Douglas

**Affiliations:** 1 Department of Molecular Biology, University of Texas Southwestern Medical Center, Dallas, TX 75390, USA; 2 O’Donnell Brain Institute, UT Southwestern Medical Center, Dallas, TX 75390, USA; 3 Hamon Center for Regenerative Science and Medicine, UT Southwestern Medical Center, Dallas, TX 75390, USA

**Keywords:** molecular neuroscience, neuroscience

## Abstract

The selective vulnerability of dopaminergic neurons to trauma-induced neurodegeneration is conserved across species, from nematodes to humans. However, the molecular mechanisms underlying this hypersensitivity to blunt force trauma remain elusive. We find that extravesicular dopamine, a key driver of Parkinson’s disease, extends its toxic role to the acute challenges associated with injury. Ectopic dopamine synthesis in serotonergic neurons sensitizes this resilient neuronal subtype to trauma-induced degeneration. While dopaminergic neurons normally maintain dopamine in a functional and benign state, trauma-induced subcellular redox imbalances elicit dopamine-dependent cytotoxicity. Cytosolic dopamine accumulation, through perturbations to its synthesis, metabolism, or packaging, is necessary and sufficient to drive neurodegeneration upon injury and during aging. Additionally, degeneration is further exacerbated by rapid upregulation of the rate-limiting enzyme in dopamine synthesis, *cat-2*, via the FOS-1 transcription factor. Fundamentally, our study in *C. elegans* unravels the molecular intricacies rendering dopaminergic neurons uniquely prone to physical perturbation across evolutionary lines.

## Highlights

• Trauma resistant neurons can be sensitized through ectopic dopamine synthesis
• Dopaminergic neurons experience excessive oxidative stress upon blunt force trauma
• Mitochondrial calcium buffering and cytosolic dopamine synergistically drive ROS
• FOS-1 dependent activation of dopamine synthesis upon trauma worsens degeneration

## Graphical abstract

**Figure:**
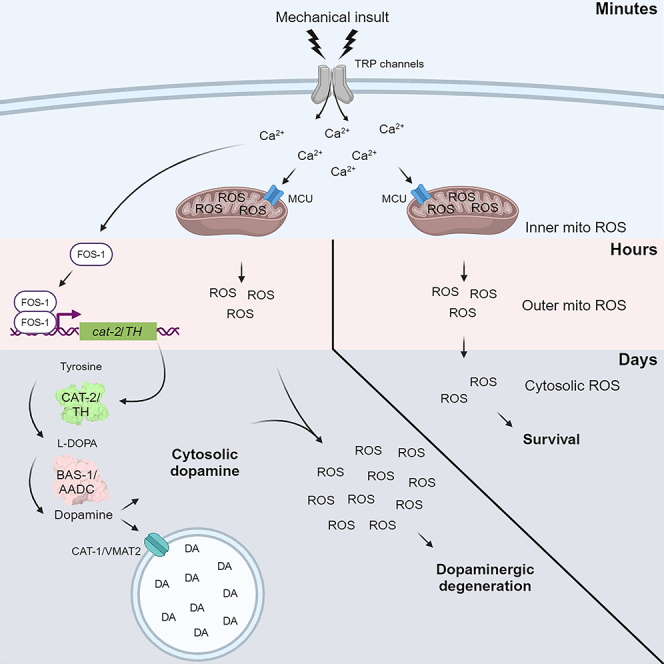

## Introduction

Dopaminergic neurons have long been recognized as particularly prone to degeneration in age-related neurodegenerative conditions like Parkinson’s disease (PD). Recently, emerging preclinical evidence has highlighted their hypersensitivity to traumatic brain injury (TBI),^1^^,^^2^^,^^3^ and substantial clinical studies have associated TBI as a significant risk factor (hazard ratio = 3.56) for developing PD and related disorders later in life.^4^^,^^5^ Strikingly, these studies also noted that both an increased time spent unconscious and increased number of sustained TBI’s proportionally increased PD risk, revealing a severity and frequency dependent relationship.^4^^,^^5^ While extensive research has focused on factors influencing dopaminergic vulnerability in PD, understanding the molecular mechanisms underlying dopaminergic degeneration due to the physical stress caused by blunt force trauma remains poorly understood.

Several theories attempt to explain the heightened vulnerability of dopaminergic neurons to cellular stress and aging. These include α-synuclein aggregation, increased energetic demand stemming from extensive axonal arborization, calcium-related pacemaking, and oxidation or metabolism of cytosolic dopamine.^6^^,^^7^^,^^8^^,^^9^^,^^10^^,^^11^^,^^12^^,^^13^ Taking an evolutionary perspective, we investigated the conservation of these traits in *C. elegans,* which is the simplest organism that exhibits selective sensitivity of dopaminergic neurons to cellular stress.^3^^,^^14^ This approach highlights that the presence of dopamine stands as the universally shared feature. Notably, *C. elegans* lack an α-synuclein counterpart and its dopaminergic neurons do not display extensive arborization or pacemaking activity. Thus, while these traits have been reported to contribute to neurodegeneration, they are not prerequisites for dopaminergic hypersensitivity.

The proximity of hydroxyl groups on dopamine’s catechol ring makes it highly susceptible to auto-oxidation within the neutral pH of the cytosol, thereby inducing a cascade of reactive oxygen species (ROS) and covalently altering intracellular components and organelles.^6^^,^^11^^,^^15^^,^^16^^,^^17^^,^^18^^,^^19^ Furthermore, cytosolic dopamine can also be metabolized by mitochondrially tethered monoamine oxidases. While this metabolism prevents dopamine quinone formation due to auto-oxidation, it has been reported to drive the electron transport chain and generate mitochondrial ROS.^13^ Fortunately, the acidic environment maintained by vacuolar H^+^ -ATPases within synaptic vesicles supports dopamine’s stability in a reduced state, enabling a means for cells to sequester dopamine away from the cytosol and averting its auto-oxidation and metabolism.^20^^,^^21^ While this delicate balance prevents cellular damage during homeostasis, disruption of proper packaging has been implicated as an important driver of dopaminergic degeneration.^7^

Utilizing a high-throughput *C. elegans* model of blunt force trauma,^14^ we explore the essential role of cytosolic dopamine in determining the selective vulnerability of dopaminergic neurons to stress and age. Additionally, we unravel the impact of trauma-induced immediate-early gene activation on disrupting dopamine homeostasis via induction of the rate-limiting enzyme of dopamine synthesis.

## Results

### Ectopic dopamine production sensitizes serotonergic neurons to trauma-induced death

**Figure 1:**
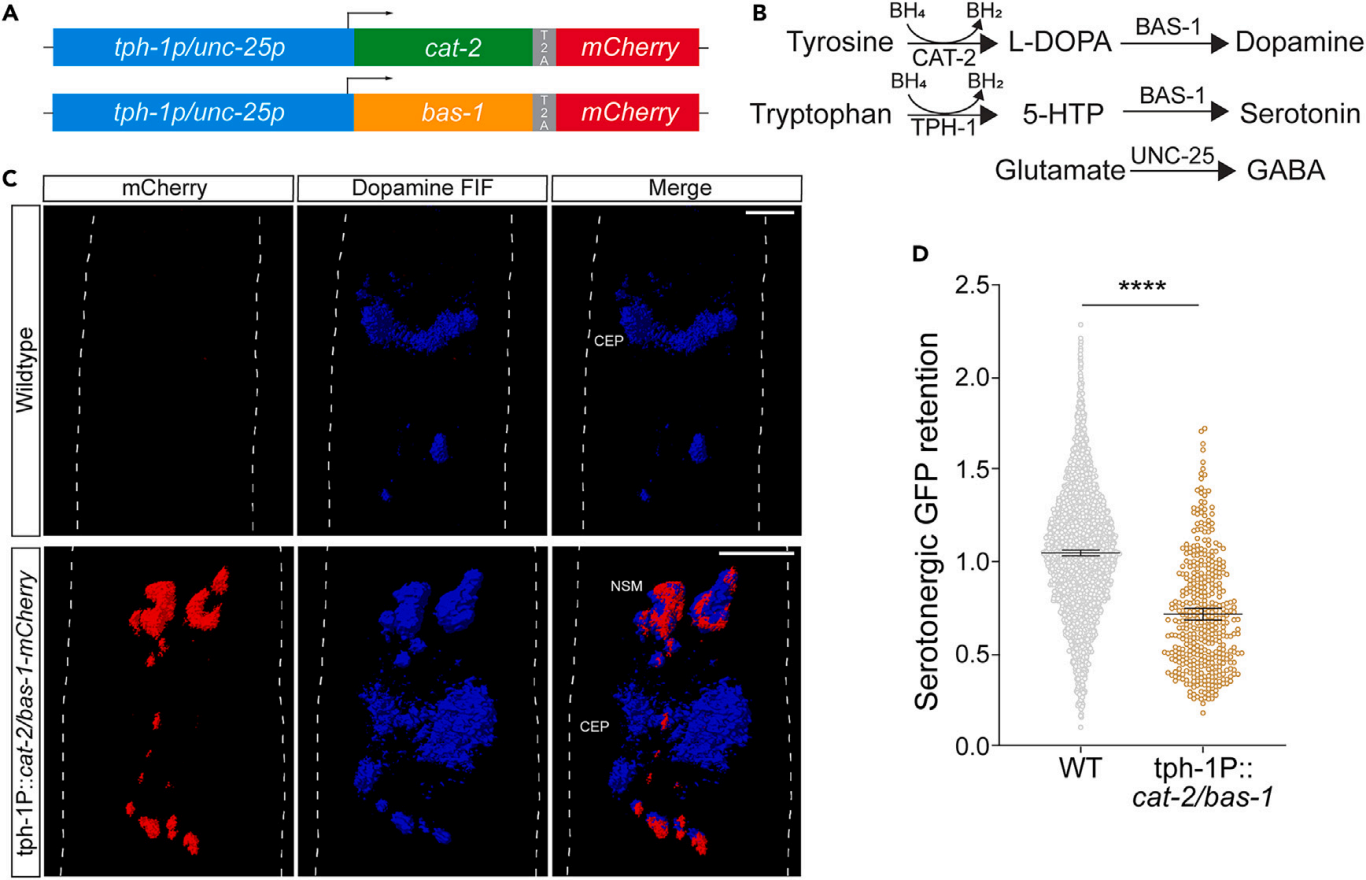
Ectopic dopamine production sensitizes serotonergic neurons to trauma-induced death (A) Schematic of expression plasmid design used to ectopically express dopamine synthesis enzymes (CAT-2 and BAS-1) in serotonergic (*tph-1p* promoter) and GABAergic (*unc-25p* promoter) neurons. Inclusion of T2A site enables co-expression of the respective transgene with mCherry. (B) Neurotransmitter biosynthetic pathways present in dopaminergic, GABAergic, and serotonergic neurons. (C) Fluorescence micrograph shows the three-dimensional renders of worm head regions compiled from z stack images. Fixed worms are stained for dopamine (blue) using formaldehyde-induced fluorescence (FIF) and compared with mCherry (red) expression. CEP annotation indicates the 4 cephalic dopamine neurons, NSM indicates two serotonergic neurons. White dashed line indicates the worm outline. Scale bar, 5 μM. (D) Serotonergic GFP retention in non-transgenic control versus transgenic worms (PMD147) ectopically expressing dopamine (*tph-1P*::*cat-2/bas-1*::*mCherry*) 48 h after injury. Each point represents an injured worm normalized to the mean of the uninjured control population. Mean ±95%CI. *n* = 4089 (WT) and 412 (*tph-1P*::*cat-2/bas-1*::*mCherry)*. Statistical analysis was performed using an unpaired t test. ∗∗∗∗ **=***p <* 0.0001.

Dopaminergic and serotonergic neurons, both being monoamine-producing subtypes, share fundamental similarities. Nevertheless, while *C. elegans’* dopaminergic neurons are highly prone to mechanical stress, serotonergic neurons demonstrate complete resilience.^3^ To investigate whether this substantial contrast could be attributed to the dopamine neurotransmitter itself, we engineered transgenic worm strains that ectopically expressed the dopamine synthesis genes *cat-2* and *bas-1* under the regulation of the serotonergic *tph-1* promoter, while using the GABAergic *unc-25* promoter as a control (Figure 1A). Only dopaminergic and serotonergic neurons possess the necessary machinery for synthesizing and recycling the tetrahydrobiopterin (BH_4_) cofactor, vital for the synthesis of their respective monoamines by *cat-2* and *tph-1* (Figures 1B and S1A).^22^ Confirmation of ectopic dopamine synthesis in serotonergic neurons was achieved through formaldehyde-induced fluorescence.^23^ As anticipated, ectopic expression of the same dopamine synthesis enzymes in GABAergic neurons failed to induce dopamine synthesis (Figures 1C and S1B). In serotonergic neurons, inducing dopamine synthesis significantly heightened their susceptibility to trauma-induced death as indicated by the loss of GFP retention two days post-injury, while wild-type serotonergic neurons did not exhibit any decrease in GFP signal (Figure 1D). Conversely, the expression of *cat-2* and *bas-1* in GABAergic neurons had only a minimal effect on GFP retention within those neurons following trauma (Figures 1C and S1D). These findings suggest that the presence of dopamine alone is sufficient to determine the susceptibility of neuronal subtypes to mechanical injury and poses an additional challenge to neurons during periods of cellular stress.

### Genetically encoded peroxide sensors reveal that dopaminergic neurons experience excessive cytosolic oxidative stress

**Figure 2:**
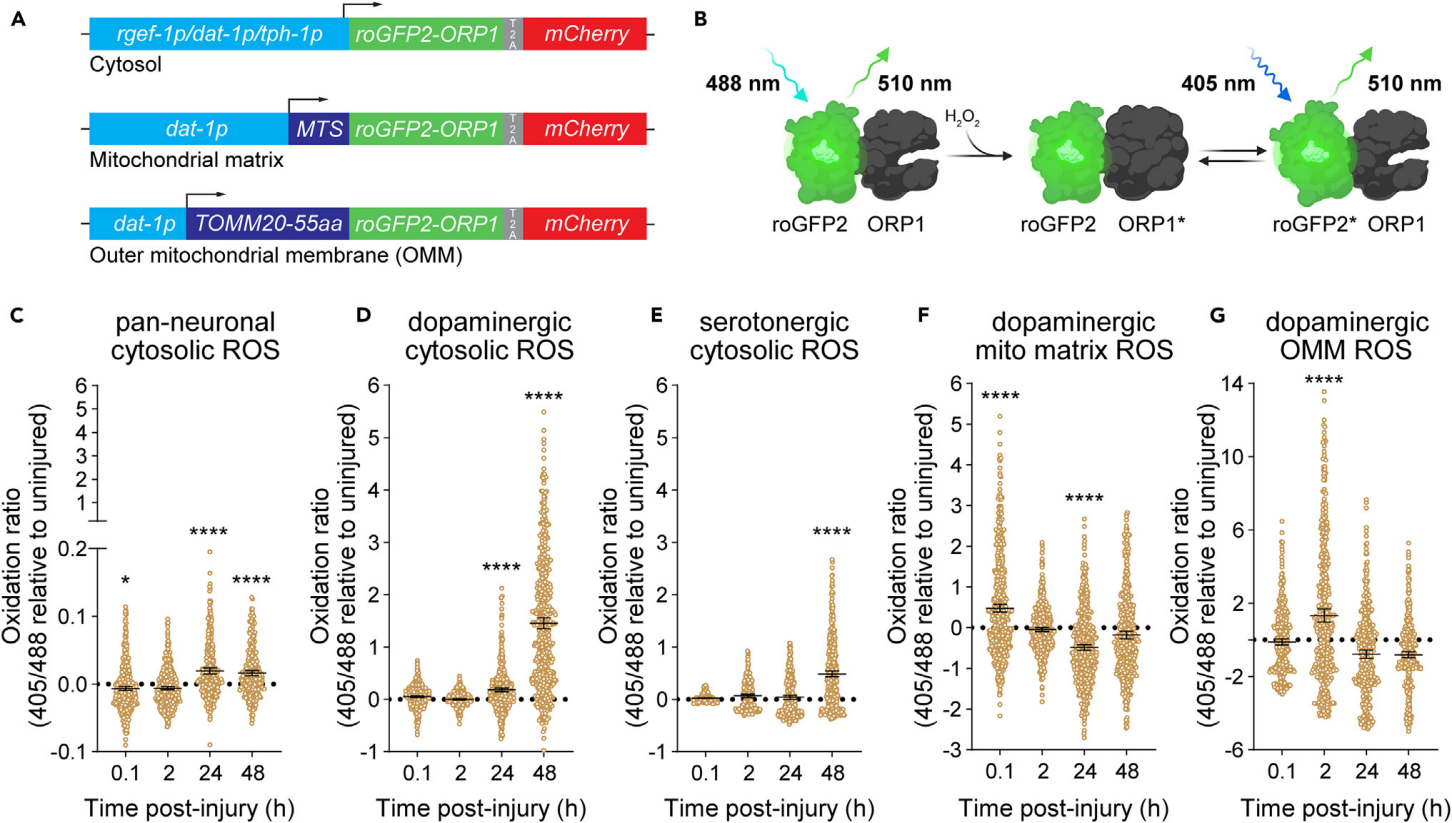
Genetically encoded peroxide sensors reveal that dopaminergic neurons experience excessive cytosolic oxidative stress (A) Schematic of expression plasmid design used to ectopically express roGFP2-ORP1 in dopaminergic (*dat-1p* promoter), serotonergic (*tph-1p* promoter) or all (*rgef-1p* promoter) neurons within the worm. MTS (mitochondrial targeting sequence) and TOMM20-55aa (first 55 amino acids of the TOMM20 outer mitochondrial translocase). Inclusion of T2A site enables co-expression of the respective transgene with mCherry. (B) Illustration of roGFP2-ORP1 ROS sensors. GFP excitation wavelength shifts from 488 nm to 405 nm upon oxidation by hydrogen peroxide. (C) Cytosolic ROS production in all head neurons after injury of roGFP2-ORP1 expressing transgenic worms (PMD184). 405 nm/488 nm oxidation ratio is normalized to uninjured, time-matched controls. Statistical analysis was performed on injured 405 nm/488 nm ratio vs. uninjured 405 nm/488 nm ratio using an ordinary one-way ANOVA with Šidák’s multiple comparisons test (see also Figure S2A). Mean ±95%CI. From left to right, n = 570, 440, 336, and 347 collected over 3 individual experiments. (D) Cytosolic ROS production in dopaminergic neurons after injury of roGFP2-ORP1 expressing transgenic worms (PMD185). 405 nm/488 nm oxidation ratio is normalized to uninjured, time-matched controls. Statistical analysis was performed on injured 405 nm/488 nm ratio versus uninjured 405 nm/488 nm ratio using an ordinary one-way ANOVA with Šidák’s multiple comparisons test (see also Figure S2B). Mean ±95%CI. From left to right, *n* = 796, 642, 526, and 552 collected over 5 independent experiments. (E) Cytosolic ROS production in serotonergic neurons after injury of roGFP2-ORP1 expressing transgenic worms (PMD188). 405 nm/488 nm oxidation ratio is normalized to uninjured, time-matched controls. Statistical analysis was performed on injured 405 nm/488 nm ratio versus uninjured 405 nm/488 nm ratio using an ordinary one-way ANOVA with Šidák’s multiple comparisons test (see also Figure S2C). Mean ±95%CI. From left to right, *n* = 997, 745, 480, and 767 collected over 3 independent experiments. (F and G) Mitochondrial-localized ROS production determined from inner (F) and outer (G) mitochondrially targeted ROS sensors within dopaminergic neurons. Time course begins with injury of day 1 adult worms. 405 nm/488 nm oxidation ratio is normalized to uninjured, time-matched controls. Statistical analysis was performed on injured versus uninjured 405 nm/488 nm ratios using an ordinary one-way ANOVA with Šidák’s corrected multiple comparisons (see also Figures S2D and S2E). Mean ±95%CI. (F) From left to right, *n* = 633, 573, 641, and 483 collected over 3 independent experiments. (G) From left to right, *n* = 450, 433, 434, and 446 collected over 3 independent experiments. ∗ **=***p <* 0.05, ∗∗ **=***p <* 0.01, ∗∗∗ **=***p <* 0.001, and ∗∗∗∗ **=***p <* 0.0001.

Upon oxidization, dopamine can form highly reactive dopamine quinones and various oxidizing intermediates while producing superoxide radicals, further perpetuating the formation of ROS through the modification of mitochondrial electron transport chain complexes and regulators of lysosomal function and mitophagy.^11^^,^^15^ Additionally, monoamine oxidase metabolism of cytosolic dopamine can drive excessive mitochondrial electron transport chain activity, resulting in mitochondrial ROS production.^13^ As dopaminergic neurons bear this additional oxidative burden, we posited that they might endure excessive oxidative stress upon injury. To delve into the subcellular redox dynamics occurring within the neuron after injury, we ectopically expressed the genetically encoded peroxide sensor, roGFP2 fused to a peroxidase (ORP1)^24^ in different subcellular locations of distinct neuronal subtypes within the worm (Figures 2A and S2A). Upon oxidation, ORP1-roGFP2 undergoes an excitation wavelength shift from 488 nm to 405 nm, enabling the quantification of cellular and subcellular redox status in living worms (Figure 2B). When expressed throughout the nervous system using the neural-specific *rgef-1* promoter, trauma induced only a modest increase in cytosolic oxidation 24 h post-injury (Figures 2C and S3A). However, exclusive expression in the cytosol of dopaminergic neurons via the *dat-1* promoter revealed a substantially larger increase in cytosolic peroxide levels at 24- and 48-h post-injury compared to pan-neuronal or serotonergic-expressed ROS sensors driven by the *tph-1* promoter (Figures 2D, 2E, S3B, and S3C). Thus, dopaminergic neurons experience disproportionately higher levels of ROS after trauma.

Intriguingly, increased oxidation in the cytosol was detectable in all neuronal subtypes tested as early as 24 h after injury. However, these redox kinetics did not align with the rapid peroxide production previously reported in whole worms occurring minutes after mechanical insult.^3^ We therefore hypothesized that trauma-induced ROS production might originate in a more compartmentalized manner before accumulating in the cytosol. Given that mitochondria are a significant source of ROS and mitochondrial dysfunction is implicated in the pathogenesis of both TBI and Parkinson’s disease,^3^^,^^25^^,^^26^ we engineered mitochondrially localized ROS probes in dopaminergic neurons (Figure S2B). Through an N-terminal localization sequence, mitochondrially targeted probes displayed elevated basal ROS levels in uninjured worms, likely due to baseline mitochondrial respiration (Figures S3D and S3E). Yet following injury, we observed an almost immediate (<5 min) rise in ROS within the mitochondria of dopaminergic neurons, resolving to baseline levels within 2 h (Figures 2F and S3D). To track the flow of intra-mitochondrial ROS to the cytosol, we directed the ORP1-roGFP2 redox probe to the outer mitochondrial membrane via roGFP2 fusion with the first 55 amino acids of the TOMM-20 translocase, possessing an outer mitochondrial membrane spanning segment. Using this construct, peak ROS formation was observed 2 h after injury but decreased below baseline pre-injury levels by 24 h (Figures 2G and S3E).

### Dopaminergic calcium influx precedes and drives trauma-induced oxidative stress

**Figure 3:**
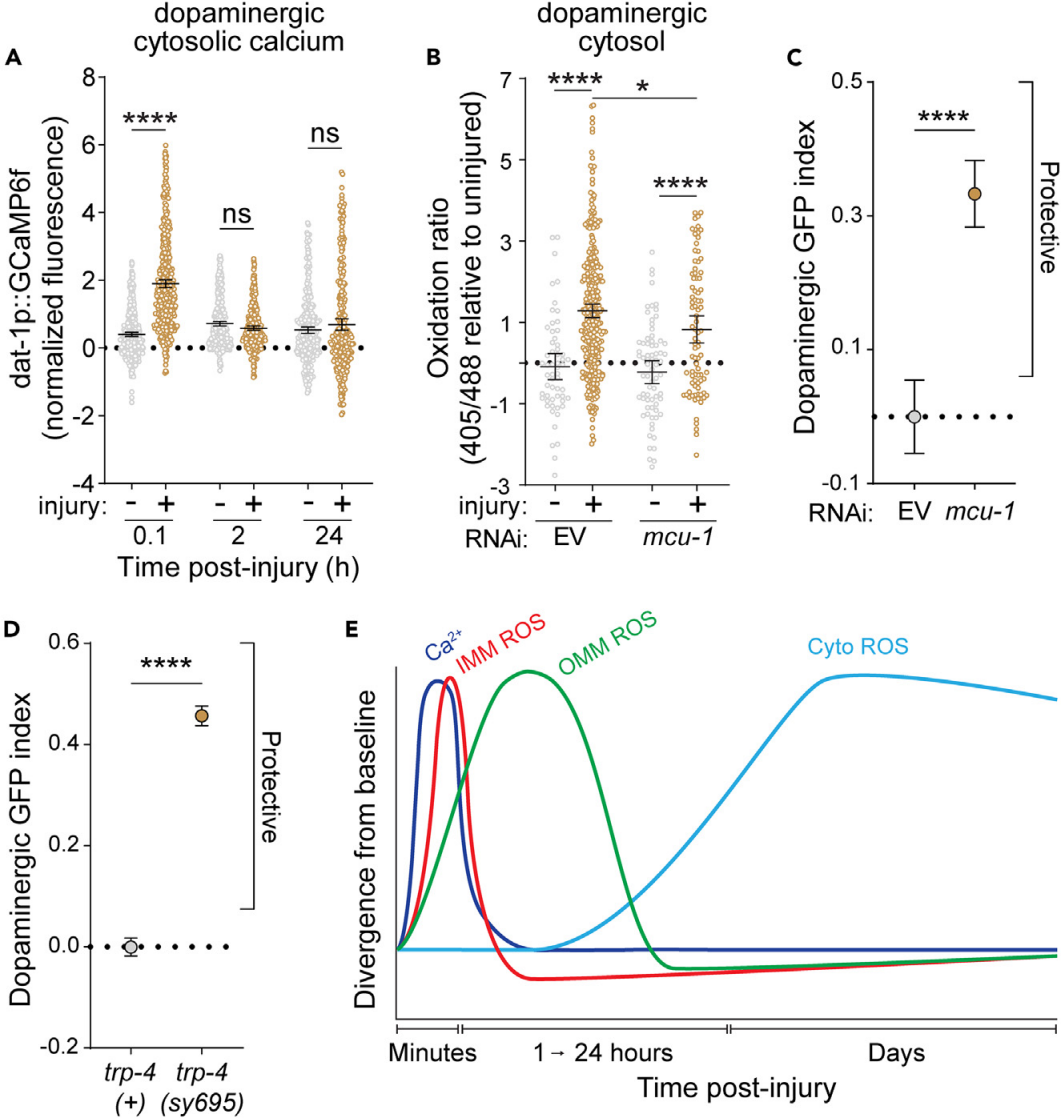
Dopaminergic calcium influx precedes and drives trauma induced oxidative stress (A) Quantification of cytosolic calcium in dopaminergic neurons after injury. GCaMP6f GFP peak height fluorescence was first normalized to non-transgenic background controls and then to time matched uninjured transgenic controls. After outlier removal, statistical analysis was performed using an ordinary one-way ANOVA with Šidák’s multiple comparisons test. Mean ±95%CI. From left to right, *n* = 475, 564, 460, 388, 412, and 307 and were collected over 3 independent experiments. (B) Cytosolic ROS production in dopaminergic neurons of roGFP2-ORP1 expressing transgenic worms (PMD249) raised on empty vector control (EV) or *mcu-1* RNAi and injured at day 1 of adulthood. ROS levels were determined 48 h after injury. All samples were normalized to the 405 nm/488 nm ratio of uninjured worms on control EV RNAi. Statistical analysis was performed using an ordinary one-way ANOVA with Šidák’s multiple comparisons test. Mean ±95%CI. From left to right, *n* = 58, 349, 73, and 87. (C) Quantification of dopaminergic GFP retention in worms (PMD216) 48 h after injury. Dopaminergic GFP index compares the loss in GFP retention upon injury in worms treated with empty vector control (EV) RNAi (injured/uninjured) to the loss in GFP retention for *mcu-1* RNAi. Positive and negative values show enhanced and reduced GFP retention respectively compared to control RNAi conditions. Statistical analysis was performed using an unpaired t test. Mean ±95%CI. *n* = 2128 (EV) and 2760 (*mcu-1*) collected over 3 independent experiments. (D) Dopaminergic GFP retention of PMD190 *(trp-4(+))* vs. PMD191 *(trp-4(sy695))* 48 h after injury raised on EV. Statistical analysis was performed using an unpaired t test. Plots show mean ±95%CI. PMD190 *n* = 4890 and PMD191 *n* = 4891 and were collected over 3 independent experiments. (E) Schematic of calcium flux and ROS accumulation over time following trauma. ∗ **=***p <* 0.05, ∗∗ **=***p <* 0.01, ∗∗∗ **=***p <* 0.001, and ∗∗∗∗ **=***p <* 0.0001.

Given the rapid kinetics observed, it is plausible that mitochondrial redox imbalances stem from injury-induced calcium flux, serving as the primary trigger within minutes. The neurometabolic cascade of concussion posits that immediate molecular events following TBI involve aberrant intraneuronal ionic fluctuations, induced by processes such as membrane deformation, axonal hyperextension, or aberrant channel activity.^27^ Specifically, disturbances in intracellular calcium ion concentration are implicated in cell damage and death post-TBI.^28^ To test if our model of mechanical stress induced any calcium flux in dopaminergic neurons, we leveraged a worm strain with dopaminergic expression of the calcium sensor GCaMP6f.^29^ Subjecting these worms to injury and assessing dopaminergic cytosolic calcium over time revealed a rapid increase of cytosolic calcium concentration within minutes after injury (Figure 3A). Similar to the observed mitochondrial matrix ROS production, this rapid increase fully recovered within 2 h.

To maintain cytosolic calcium at a minimum, calcium is rapidly taken up by the endoplasmic reticulum and mitochondria as the main calcium stores in the cell. The principal mechanism employed by mitochondria to buffer calcium levels is through the mitochondrial calcium uniporter, *mcu-1*. However, an overload of mitochondrial calcium can instigate ROS formation. Our investigation focused on discerning the role of mitochondrial calcium overload in post-injury scenarios and its potential correlation with ROS formation in dopamine neurons. To explore this, we sought to perturb MCU-1 and measure injury-induced ROS. Since *C. elegans* neurons are typically refractory to dsRNAi feeding, we employed a strategy involving the crossing of worms expressing the cytosolically localized dopaminergic ROS sensor with a strain possessing a *dat-1* driven overexpression of RNAi uptake and processing genes *sid-1* and *rde-1*, respectively. Notably, this genetic manipulation was executed in a *rde-1* null background, thereby exclusively targeting the RNAi to dopaminergic neurons. Administration of *mcu-1* RNAi to these worms, coupled with subsequent injury, yielded a significant reduction in cytosolic ROS within dopamine neurons 48 h post-injury (Figure 3B). However, ROS production exhibited only a partial reduction, suggesting the involvement of other factors in the genesis of injury-induced ROS formation. Intriguingly, this modest reduction of cytosolic ROS proved sufficient to protect dopaminergic neurons from trauma-induced degeneration as evidenced by an increased dopaminergic GFP index (Figure 3C).

To bolster the involvement of aberrant calcium flux being an early trigger of dopaminergic degeneration, we wondered if perturbing this influx further upstream in the cascade would be protective as well. Based on the literature, a likely source of mechanical stress induced calcium flux in *C. elegans* dopamine neurons is the TRP-4 channel. TRP-4 is a TRPN-type transient receptor potential mechanosensory channel and gain-of-function mutations have been implicated in dopaminergic degeneration.^30^ To test whether TRP-4 contributes to trauma-induced dopaminergic degeneration, we crossed *trp-4* mutant worms, TQ296*(trp-4(sy695))*, with our PMD13 dopaminergic GFP strain. From this cross, we isolated both homozygous *trp-4* mutants and homozygous wild types as genetic background controls. Subjecting both of these worm strains to injury revealed that *trp-4* mutants had significantly increased dopaminergic GFP retention (Figure 3D).

Taken together, excessive calcium influx appears to be one of the earliest and most upstream molecular consequences of trauma. This increased cytosolic calcium is rapidly buffered, in part by mitochondria, which can drive mitochondrial ROS production. Eventually, this mitochondrial ROS appears to “spill-out” into the cytosol which persists as a long-term physiological consequence of the initial insult (Figure 3E). Remarkably, while perturbation of mitochondrial calcium influx is sufficient to protect, it is only partially responsible for total dopaminergic cytosolic ROS production.

### Cytosolic dopamine drives dopaminergic oxidative stress and degeneration

**Figure 4:**
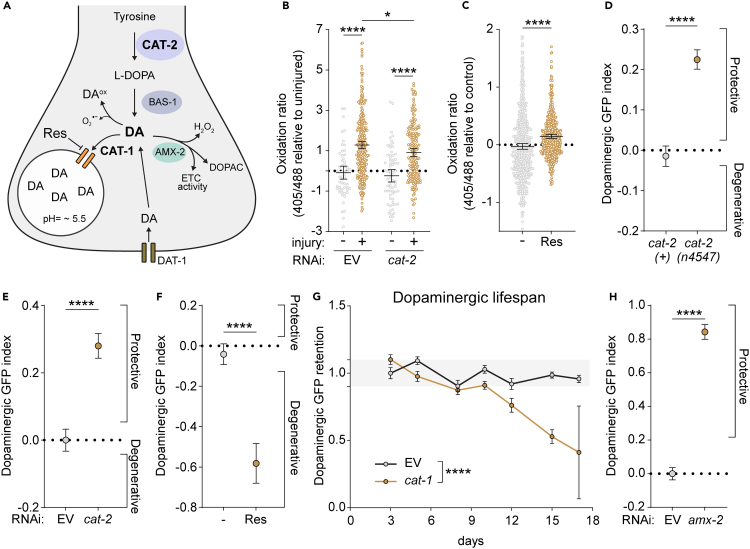
Cytosolic dopamine drives dopaminergic oxidative stress and degeneration (A) Schematic of dopamine (DA) homeostasis in the synapse of *C. elegans* dopaminergic neurons. CAT-2 = tyrosine hydroxylase, BAS-1 = aromatic amino acid decarboxylase, DA = dopamine, DA^ox^ = dopamine oxidation products, AMX-2 = monoamine oxidase, CAT-1 = vesicular monoamine transporter 2, DAT-1 = dopamine reuptake transporter. (B) Cytosolic ROS production in dopaminergic neurons of worms (PMD249) raised on empty vector control (EV) or *cat-2* RNAi and injured at day 1 of adulthood. Dopaminergic cytosolic ROS was determined 48 h after injury. Blank normalized 405 nm/488 nm ratios were used for statistical analysis using an ordinary one-way ANOVA with Šidák’s multiple comparisons test. Gray = uninjured, gold = injured. Mean ±95%CI. From left to right, *n* = 58, 349, 70, and 200. (C) Cytosolic ROS production in dopaminergic neurons of uninjured worms (PMD185). 405 nm/488 nm oxidation ratio is normalized to vehicle control (acetic acid). Reserpine (Res) was supplemented in agar plates at a final concentration of 30 μM for 2 days starting from day 1 of adulthood. Statistical analysis was performed using an ordinary one-way ANOVA with Dunnett’s multiple comparisons test. Blue = uninjured, beige = injured. Mean ±95%CI. From left to right, *n* = 585 and 671 collected over 3 independent experiments. (D) Quantification of dopaminergic GFP retention in wild type (PMD13) or *cat-2(n4547)* mutant worms (PMD156) 48 h after injury. Dopaminergic GFP index compares the loss in GFP retention upon injury in the control population (injured/uninjured) to the loss in GFP retention for *cat-2(n4547)* mutants. Positive and negative values show enhanced and reduced GFP retention respectively compared to wild-type conditions. Statistical analysis was performed using an unpaired t test. Mean ±95% CI. *n* = 7540 (*cat-2(+))* and 8915 *(cat-2(n4547))* collected over 3 independent experiments. (E) Quantification of dopaminergic GFP retention in worms (PMD216) 48 h after injury. Dopaminergic GFP index compares the loss in GFP retention upon injury in worms treated with *cat-2* RNAi to the loss in GFP retention for empty vector control (EV) RNAi (injured/uninjured). Positive and negative values show enhanced and reduced GFP retention respectively compared to EV control RNAi conditions. Statistical analysis was performed using an unpaired t test. Mean ±95% CI. From left to right, *n* = 3696 and 3585 collected over 4 independent experiments. (F) Quantification of dopaminergic GFP retention in worms (PMD216) 48 h after injury, treated with 30 μM reserpine (Res) or vehicle control (acetic acid) post-injury. Dopaminergic GFP index compares the loss in GFP retention upon injury in worms treated with vehicle control (injured/uninjured) to the loss in GFP retention for the respective reserpine treatment regiments. Positive and negative values show enhanced and reduced GFP retention respectively compared to vehicle control. Statistical analysis was performed using an ordinary one-way ANOVA with Dunnett’s multiple comparisons test. Mean ±95%CI. From left to right, *n* = 1828 and 1694 collected over 3 independent experiments. (G) Quantification of dopaminergic GFP retention in worms (PMD216) maintained on EV or *cat-1* RNAi. Raw GFP fluorescence across all timepoints are normalized to EV at day 3 of adulthood. Statistical analysis was performed using an ordinary two-way ANOVA comparing the column factor between EV and *cat-1* RNAi over multiple time points. Mean ±95%CI. From left to right, EV *n* = 424, 944, 294, 1043, 519, 1507, 1075. *cat-1 n*= 569, 418, 544, 968, 304, 136, 7. (H) Quantification of dopaminergic GFP retention in worms (PMD216) 48 h after injury. Dopaminergic GFP index compares the loss in GFP retention upon injury in worms treated with empty vector control (EV) RNAi (injured/uninjured) to the loss in GFP retention for the respective RNAi. Positive and negative values show enhanced and reduced GFP retention respectively compared to EV control RNAi conditions. Statistical analysis was performed using an unpaired t test. Mean ±95% CI. From left to right, *n* = 3770 and 3389 collected over 3 independent experiments. ∗ **=***p <* 0.05, ∗∗ **=***p <* 0.01, ∗∗∗ **=***p <* 0.001, and ∗∗∗∗ **=***p <* 0.0001.

To investigate dopamine’s contribution in cytosolic ROS formation, we aimed to manipulate dopamine synthesis through *cat-2* knockdown and assess injury-induced oxidative stress. Worms raised on *cat-2* RNAi and subjected to injury exhibited a discernable reduction in cytosolic ROS levels in dopaminergic neurons 48 h post-injury compared to those raised on empty vector control (EV) (Figure 4B). This reduction was comparable in magnitude to that observed with *mcu-1* RNAi implying an equivalent impact of mitochondrial calcium overload and dopamine on the induction of oxidative stress following injury. Additionally, in the absence of injury, administering the dopamine packaging inhibitor reserpine to healthy adult worms for two days led to formation of cytosolic ROS (Figure 4C). Subsequently, we delved into the physiological implications of these redox changes on neurodegeneration. Mutants lacking dopamine synthesis, *cat-2(n4547)*, and worms subjected to *cat-2* RNAi both exhibited reduced dopaminergic degeneration after trauma, evidenced by increased dopaminergic GFP retention and an elevated dopamine GFP index (Figures 4D and 4E). Conversely, augmenting cytosolic dopamine post-insult via reserpine supplementation significantly exacerbated dopaminergic degeneration (Figures 4A and 4F). Wondering how a prolonged increase in cytosolic dopamine might impact age-related neuronal loss, we employed selective RNAi exclusively in *dat-1* positive neurons and subjected *C. elegans* to *cat-1* RNAi from hatching until day 17 of adulthood. Assessing dopaminergic GFP fluorescence every 2–3 days revealed a substantial loss of GFP retention after 12 days of aging in *cat-1* RNAi fed animals as compared to control (Figure 4G). Thus, perturbing dopamine packaging can accelerate age-related loss of dopaminergic neurons.

Since extravesicular dopamine can be toxic through both its auto-oxidation and its metabolism (Figure 4A), we sought to investigate the route of toxicity. To test this, we perturbed dopamine metabolism by monoamine oxidases through *amx-2* RNAi.^31^ If cytosolic dopamine were predominantly toxic through its auto-oxidation, *amx-2* RNAi would worsen trauma-induced degeneration. Reciprocally, if monoamine oxidase metabolism was the primary toxic route, *amx-2* RNAi would have a net protective effect. Dopaminergic exclusive knockdown of *amx-2* confirmed the latter as dopaminergic neurons lost significantly less GFP following injury when raised on *amx-2* RNAi (Figure 4H).

Further investigation was conducted to ascertain whether increased cytosolic dopamine triggered cellular stress independent of reduced dopamine release. To discern this, we compared genes differentially expressed upon dopaminergic *cat-1* RNAi, excluding those altered by *cat-2* RNAi, thereby enriching for genes responding to increased cytosolic dopamine while subtracting genes influenced by reduced dopamine release. This approach highlighted differential regulation of recognized stress response genes and several unassigned genes annotated to be responsive to cellular stress (Figures 3C and S4A). Further emphasizing how elevated cytosolic dopamine can act as a deleterious stimulus to the cell, independent of its neurotransmissive function. Lastly, gene set enrichment analysis of *cat-1* RNAi responsive yet *cat-2* RNAi resistant genes found an enrichment of genes involved in the cellular response to calcium and metal ions, and hydrogen peroxide metabolic and catabolic processes (Figure S4D).

### Trauma-induced immediate-early gene activation promotes dopamine synthesis and worsens dopaminergic degeneration

**Figure 5:**
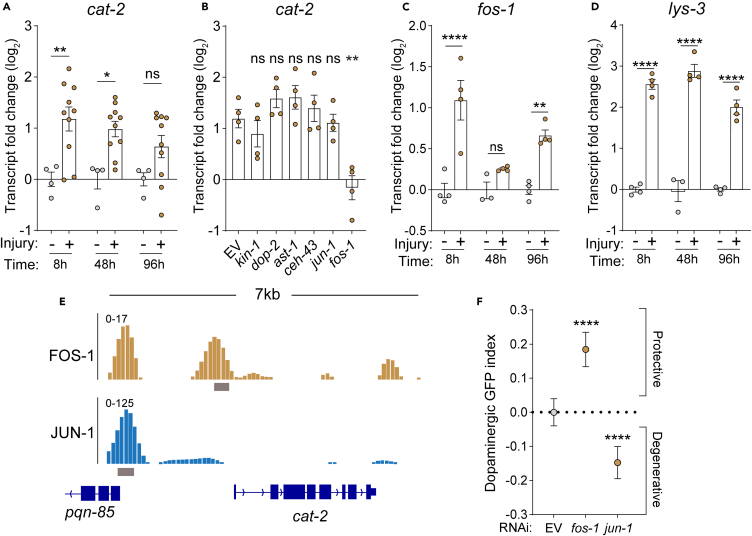
Trauma-induced immediate early gene activation promotes dopamine synthesis and worsens dopaminergic degeneration (A) RT-qPCR quantification of *cat-2* fold change of CF512 worms injured at day 1 of adulthood normalized to age-matched uninjured controls. Statistical analysis was performed using an ordinary one-way ANOVA with Dunnet’s multiple comparisons test. *n* = 4 uninjured and 10 injured independent replicates. (B) RT-qPCR quantification of *cat-2* fold change of PMD63 worms 48 h post-injury normalized to age-matched uninjured controls on respective RNAi. Statistical analysis was performed using an ordinary one-way ANOVA with Dunnet’s multiple comparisons test. *n* = 4 independent replicates per condition. (C) RNA-seq quantification of *fos-1* in CF512 worms injured at day 1 of adulthood and collected 8-, 48-, and 96-h post-trauma. RPKMs are normalized to age-matched uninjured controls. Statistical analysis was performed using an ordinary one-way ANOVA with Dunnet’s multiple comparisons test. *n* = 4 independent replicates per condition. (D) RNA-seq quantification of *lys-3* in CF512 worms injured at day 1 of adulthood and collected 8-, 48-, and 96-h post-trauma. RPKMs are normalized to age-matched uninjured controls. Statistical analysis was performed using an ordinary one-way ANOVA with Dunnet’s multiple comparisons test. *n* = 4 independent replicates per condition. (E) Integrative Genomics Viewer (IGV) tracks of FOS-1 (ENCODE: ENCFF583XRV and ENCFF235JBS) and JUN-1 (ENCODE: ENCFF902YYC and ENCFF225PZG) ChIP-seq in *C. elegans* L4 larvae. Tracks display control normalized signal of pooled replicates (top) and optimal IDR thresholded peaks (bottom). (F) Quantification of dopaminergic GFP retention in worms (PMD63) 48 h after injury. Dopaminergic GFP index compares the loss in GFP retention upon injury in worms treated with empty vector control (EV) RNAi (injured/uninjured) to the loss in GFP retention for the respective RNAi. Positive and negative values show enhanced and reduced GFP retention respectively compared to EV control RNAi conditions. Statistical analysis was performed using an ordinary one-way ANOVA with Dunnett’s multiple comparisons test. Mean ±95% CI. From left to right, *n* = 1418, 1269, and 1097 collected over 3 independent experiments. ∗ **=***p <* 0.05, ∗∗ **=***p <* 0.01, ∗∗∗ **=***p <* 0.001, and ∗∗∗∗ **=***p <* 0.0001.

Since healthy uninjured dopaminergic neurons do not undergo spontaneous degeneration (Figure 4G), we postulated that mechanical stress affects dopamine homeostasis. Notably, we observed increased *cat-2* transcription 8- and 24-h post-injury (Figure 5A). Since this could explain how injury disrupts dopamine homeostasis via increased synthesis, we set out to identify potential regulators of this induction. To generate a list of transcription factors (TFs) for a candidate-based screen in *C. elegans*, we leveraged neurotrauma datasets from multiple species and applied specific filtering criteria to identify TFs that might influence this response (Figure S5A). Initially, we identified TFs with significantly altered transcriptional programs in murine TBI datasets.^3^ Next, we ascertained that these TFs possessed predicted consensus binding sequences within the 5′ untranslated region (UTR) of tyrosine hydroxylase. Lastly, we ensured that these TFs had orthologues in *C. elegans* that were expressed in dopaminergic neurons according to single-cell sequencing worm database.^22^ This rigorous filtering process led to the identification of a small subset of TFs (∼15), with AP1, AP4, and ETS1 showing the largest enrichment of predicted binding sites (Figure S5B). Via neuronal-specific RNAi of these transcriptional complexes and other known regulators of *cat-2*, we discerned that the AP1 subunit, *fos-1*, was necessary for trauma-induced *cat-2* transcription, whereas the complementary AP1 subunit *jun-1* was not involved (Figure 5B). Furthermore, other established regulators of dopamine signaling such as *kin-1*, the autoreceptor *dop-2*, or master regulators of dopaminergic development *ast-1* and *ceh-43*^32^^,^^33^ were dispensable for this transcriptional regulation upon trauma (Figure 5B).

The kinetics of injury-induced *cat-2* transcription aligned with genome-wide transcriptomics from injured animals, showing transcriptional induction of *fos-1* itself and the known AP1 target gene *lys-*3 (Figure 5C and 5D). Analysis of *fos-1* and *jun-1* ENCODE chip-seq data revealed significant binding of *fos-1*, but not *jun-1*, within 500 bp of the *cat-2* transcription start site, suggesting direct regulation of *cat-2* by *fos-1* (Figure 5E). Consistent with this differential AP1 subunit requirement for *cat-2* transcription, neuronal RNAi of either subunit exhibited opposing effects, with *jun-1* RNAi exacerbating and *fos-1* RNAi suppressing trauma-induced degeneration of dopamine neurons (Figure 5F). Our previous work showed that activation of the MAPK phosphatase, *vhp-1*, after trauma required *jun-1* but not *fos-1*,^14^ which are indeed activated by trauma with opposing kinetics (Figure S5C). Taken together with this current study, this highlights how different homo- or heterodimeric combinations of these AP1 subunits elicit distinct transcriptional signatures, *jun-1* protective yet *fos-1* deleterious.

Our findings indicate that trauma-induced ionic fluctuations promote a degenerative transcriptional response through *fos-1*, involving *cat-2* activation. Based on the detrimental role of dopamine described earlier, this transcriptional induction of dopamine synthesis elucidates how blunt force injury disrupts dopamine homeostasis and exacerbates the cellular stress experienced by dopaminergic neurons.

## Discussion

Our investigation provides a mechanistic explanation for the heightened vulnerability of dopaminergic neurons to blunt-force trauma. Analogous to the pathophysiology of PD, cytosolic dopamine emerges as a pivotal factor, proving both necessary and sufficient in determining hypersensitivity to injury. We substantiate this assertion by forcing serotonergic neurons, akin to dopamine neurons, to ectopically synthesize dopamine. The outcome is hypersensitization of serotonergic neurons to trauma-induced degeneration, a phenomenon to which they are normally resilient.^3^ Overexpression of the same enzymes in GABAergic neurons, which lack the BH4 cofactor required for dopamine synthesis, did not exacerbate injury-induced degeneration to a similar extent. This observation implies that hypersensitization is attributed to dopamine synthesis rather than the overexpression of exogenous proteins.

Oxidative stress, a central feature in the demise of dopaminergic neurons in PD and TBI, plays a pivotal role in our model of mechanical stress. When subjected to this stress, dopaminergic neurons experience disproportionally more ROS compared to other neuronal subtypes. Although the precise contributing factors have been the subject of prolonged debate, our study indicates that cytosolic to mitochondrial calcium dynamics and dopamine exert nearly equal influences. The perturbation of either of these elements is individually sufficient to protect dopamine neurons from injury, underscoring a synergistic interplay between mitochondrial dysfunction and dopamine. This is further supported by the observation that serotonergic neurons, while still experiencing significant ROS formation, remain resilient to injury until the introduction of dopamine. The covalent alteration of mitochondrial proteins by oxidized dopamine^16^^,^^17^^,^^19^ and the role of dopamine metabolism driving mitochondrial ROS,^13^ lends credence to the notion that dopamine plays a direct role in oxidative stress. Future studies are required to define what routes of toxicity are predominant in disease states and whether these mechanisms are shared among PD and TBI.

Moreover, our investigation reveals that promoting the extravesicular accumulation of dopamine in the cytosol via inhibition of its vesicular packaging is sufficient to induce cytosolic ROS and drive dopaminergic degeneration induced by acute or chronic stress, i.e., trauma or aging. This ROS formation may emanate directly from dopamine oxidation or indirectly from mitochondrial damage inflicted by dopamine and dopamine metabolism. Indeed, understanding the link between acute changes in dopamine homeostasis following TBI and its potential contribution to neurodegeneration is crucial for developing effective therapeutic strategies. While our study primarily focused on the immediate effects of TBI on dopamine levels, we anticipate that these short-term alterations may have long-term consequences on neuronal health. Neurodegeneration is a complex process influenced by various factors and it is plausible that the initial disruption in cytosolic dopamine and eventual ROS production post-TBI could exacerbate these processes. Effectively, acute insults early in life might sensitize cells to progressive neuronal damage and eventual neurodegeneration induced by chronic stress through the toxic cascades described in this study.

Despite the long-standing recognition of dopamine’s potential reactivity dating back to 1976,^21^ the precise reasons for the disruption of dopamine homeostasis under stressful conditions have remained elusive. This prompted our investigation into dopamine synthesis following trauma, where we revealed the aberrant induction of the rate-limiting enzyme in dopamine synthesis by *fos-1*. This linkage between calcium dynamics and dopamine is reinforced by the positioning of *fos-1* downstream of the MAPK cascade,^34^ aligning our earlier findings of phosphorylation of ERK/MPK-1, p38/PMK-1, and JNK/KGB-1 within minutes after injury.^14^ The temporal kinetics described herein also complement our understanding of MAPK pathway activation in response to head trauma in mammalian models.^35^

Multiple preclinical and clinical studies further substantiate dopaminergic dysfunction following TBI, revealing a short-term hyperdopaminergic state followed by a protracted hypodopaminergic state following TBI (reviewed by Jenkins P.O.^36^). While the origin of cytosolic calcium warrants further investigation, the theoretical underpinnings suggest multiple avenues through which mechanical stress could induce calcium flux. As shown here, mechanosensory receptors can contribute to degeneration, yet calcium stores within the ER, and microtears in the cell membrane, given the elevation of extracellular calcium relative to cytosolic levels, all emerge as additional sources of uncontrolled ionic influx.

In summary, the molecular insights provided in this study shed light on the intricate interplay of stress-induced cytosolic calcium as a dual catalyst for dopaminergic degeneration, propelling both mitochondrial oxidative stress and increased dopamine synthesis. Consequently, our findings posit dopamine toxicity not only as a hallmark of PD-related pathologies but also as a crucial consideration for future research in the realm of TBI.

### Limitations of the study

For assessing redox imbalances in neurons in a cell-type-specific manner, we are limited to using genetically encoded ROS sensors. These probes are potentially affected by endogenous molecules and local pH, and they could be limited in their dynamic range and specificity. Furthermore, since dopaminergic neurons comprise only 8 cells out of the 959 somatic cells in the entire nematode, we are unable to biochemically verify findings in a cell-type-specific manner.

## STAR★Methods

### Key resources table

**Table:** 

REAGENT or RESOURCE	SOURCE	IDENTIFIER
**Bacterial and virus strains**		

HT115 (DE3) Escherichia coli	Rual et al.^35^	CGC Cat#HT115 (DE3), RRID: WB-STRAIN: HT115(DE3)
OP50 Escherichia coli		CGC Cat#OP50, RRID: WB-STRAIN: OP50

**Chemicals, peptides, and recombinant proteins**		

16% Paraformaldehyde, EM grade	Electron Microscopy Sciences	Cat#15710
Levamisole, 99%	Acros Organic	Cat#187870100
COPAS GP sheath reagent	Union Biometrica	Cat#300-5070-100
TRIzol	Thermo Fisher Scientific	Cat#15-596-026
Halocarbon 700 oil	Sigma Aldrich	Cat#H8898

**Critical commercial assays**		

QuantiTect Reverse Transcription kit	Qiagen	Cat#205311
iTaq Universal SYBR Green Supermix kit	Bio-Red	Cat#1525125

**Deposited data**		

Chromatin immunoprecipitation sequencing for FOS-1	ENCODE	ENCFF583XRV, ENCFF235JBS
Chromatin immunoprecipitation sequencing for JUN-1	ENCODE	ENCFF902YYC, ENCFF225PZG
Raw RNA sequencing data (N2 injured vs non-injured timecourse)	This paper	GeoDatasets: GSE254141
Raw RNA sequencing data (PMD216 on EV, cat-1, cat-2 RNAi)	This paper	GeoDatasets: GSE254138

**Experimental models: Organisms/strains**		

*C. elegans:* N2 (ancestral) as wildtype (WT)	Caenorhabditis Genetics Center	CGC Cat#N2 (ancestral), RRID: WB-STRAIN: N2_(ancestral)
C. elegans: CF512 : *rrf-3(b26) II; fem-1(hc17) IV.*	Caenorhabditis Genetics Center	CGC Cat#CF512, RRID: WB-STRAIN: CF512
C. elegans: WM27 (*rde-1(ne219) V*)	Caenorhabditis Genetics Center	CGC Cat#WM27, RRID: WB-STRAIN: WM27
C. elegans: XE1474 (*dat-1p::rde-1::SL2::sid-1 + unc-119(+); wpSi6 II; eri-1(mg366) IV; rde-1(ne219) V; lin-15B(n744) X*)	Caenorhabditis Genetics Center	CGC Cat#XE1474, RRID: WB-STRAIN: XE1474
*C. elegans:* PMD13 (*rrf-3(b26) II; fem-1(hc17) IV; egIs1 [dat-1p::GFP]*)	Egge et al.^13^	N/A
*C. elegans:* PMD14 (*rrf-3(b26) II; fem-1(hc17) IV; juIs76 [unc-25p::GFP* + *lin-15(+)] II*)	Egge et al.^13^	N/A
*C. elegans:* PMD25 (*mgIs42 [tph-1::GFP + rol-6(su1006)]; CF512 [fer-15(b26) II; fem-1(hc17ts) IV]*)	Egge et al.^13^	N/A
*C. elegans:* PMD63 (*rrf-3(b26) II; fem-1(hc17) IV; egIs1 [dat-1p::GFP]; sid-1(pk3321) V; uIs69 [pCFJ90 (myo-2p::mCherry) + unc-119p::sid-1]*)	Egge et al.^13^	N/A
*C. elegans:* PMD184 (utsEx18 *[rgef-1p::ORP1::roGFP2::T2A::mCherry::unc-54 3’ UTR]*)	This paper	N/A
*C. elegans:* PMD185 (utsEx19 *[dat-1p::ORP1::roGFP2::T2A::mCherry::unc-54 3’ UTR]*)	This paper	N/A
C. elegans: PMD188 (utsEx22 *[tph-1p::ORP1::roGFP2::T2A::mCherry::unc-54 3’ UTR]*)	This paper	N/A
*C. elegans:* PMD189 (utsEx23 *[dat-1p::MTS::ORP1::roGFP2::T2A::mCherry::unc-54 3’ UTR]*)	This paper	N/A
*C. elegans:* PMD227 (utsEx44 *[dat-1p::55aaTOMM20::ORP1::roGFP2::T2A::mCherry::unc-54 3’ UTR])*	This paper	N/A
*C. elegans:* PMD249 (utsEx19 *[dat-1p::ORP1::roGFP2::T2A::mCherry::unc-54 3’ UTR]; wpSi6 [dat-1p::rde-1::SL2::sid-1 + unc-119(+)] II; eri-1(mg366) IV; rde-1(ne219) V; lin-15B(n744) X.)* was made by crossing PMD185 (utsEx19 *[dat-1p::ORP1::roGFP2::T2A::mCherry::unc-54 3’ UTR]*) with XE1474 (*dat-1p::rde-1::SL2::sid-1 + unc-119(+); wpSi6 II; eri-1(mg366) IV; rde-1(ne219) V; lin-15B(n744) X*).	This paper	N/A
*C. elegans:* PMD238 (utsEx16 *[unc-25p::CAT-2::mCherry::unc-54 3’ UTR; unc-25p::BAS-1::mCherry::unc-54 3’ UTR]*) and was crossed with PMD14 (*rrf-3(b26) II; fem-1(hc17) IV; juIs76 [unc-25p::GFP* + *lin-15(+)] II*) to make PMD146 (utsEx16 *[unc-25p::CAT-2::mCherry::unc-54 3’ UTR; unc-25p::BAS-1::mCherry::unc-54 3’ UTR]; rrf-3(b26) II; fem-1(hc17) IV; juIs76 [unc-25p::GFP* + *lin-15(+)] II)]*)	This paper	N/A
*C. elegans:* PMD239 (utsEx17 *[tph-1p::CAT-2::mCherry::unc-54 3’ UTR; tph-1p::BAS-1::mCherry::unc-54 3’ UTR]*) and was crossed with PMD25 (*mgIs42 [tph-1::GFP + rol-6(su1006)]; CF512 [rrf-3(b26) II; fem-1(hc17ts) IV]*) to make PMD147 (utsEx17 *[tph-1p::CAT-2::mCherry::unc-54 3’ UTR; tph-1p::BAS-1::mCherry::unc-54 3’ UTR]; mgIs42 [tph-1::GFP + rol-6(su1006)] rrf-3(b26) II; fem-1(hc17ts) IV*)	This paper	N/A
*C. elegans:* PMD216 (*fem-1(hc17) IV, rrf-3(b26) II; egIs1 [dat-1p::GFP]; dat-1p::rde-1::SL2::sid-1 + unc-119(+); wpSi6 II; eri-1(mg366) IV; rde-1(ne219) V; lin-15B(n744) X*) was made by crossing PMD13 (*rrf-3(b26) II; egIs1 [dat-1p::GFP]*) with XE1474 (*dat-1p::rde-1::SL2::sid-1 + unc-119(+); wpSi6 II; eri-1(mg366) IV; rde-1(ne219) V; lin-15B(n744) X*).	This paper	N/A
*C. elegans:* PMD156 (*fem-1(hc17) IV, rrf-3(b26) II; egIs1 [dat-1p::GFP], cat-2(n4547) II*) was made by crossing PMD13 with MT15620 (*cat-2(n4547) II.*)	This paper	N/A
*C. elegans:* PMD191 *(fem-1(hc17) IV, rrf-3(b26) II; egIs1 [dat-1p::GFP], trp-4(sy695) I)* was made by crossing *PMD13 (rrf-3(b26) II; egIs1 [dat-1p::GFP])* and TQ296 *(trp-4(sy695) I)*	This paper	N/A
*C. elegans:* PMD190 *(fem-1(hc17) IV, rrf-3(b26) II; egIs1 [dat-1p::GFP])* was made by crossing PMD13 *(rrf-3(b26) II; egIs1 [dat-1p::GFP])* and TQ296 *(trp-4(sy695) I)* but selected for trp-4 WT allele to use as a control for PMD191.	This paper	N/A

**Oligonucleotides**		

qPCR primers, see Table S1	This paper	N/A
Genotyping primers, see Table S1	This paper	N/A
Primers to clone roGFP-ORP1 under control of dat-1, tph-1 or rgef-1 promoters, see Table S1	This paper	N/A
Primers to clone CAT-2 and BAS-1 under control of tph-1 or unc-52 promoters, see Table S1	This paper	N/A
Primers for linearizing plasmids for worm microinjections, see Table S1	This paper	N/A
RNAi sequencing, see Table S2	Vidal/Ahringer library	N/A

**Software and algorithms**		

Leica Application Suite X (LAS X) version 3.5.5	Leica	https://www.leica-microsystems.com/products/microscope-software/p/leica-las-x-ls/
LAS X topological 3D visualization tool	Leica	https://www.leica-microsystems.com/products/microscope-software/p/leica-las-af-3d-visualization/
Fiji Software	ImageJ	https://imagej.net/software/fiji/
FlowPilot Software (version 1.6.1.8)	Union Biometrica	https://www.unionbio.com/copas/
CLC Genomics Workbench (version 9.5)	Qiagen Bioinformatics	https://digitalinsights.qiagen.com/products-overview/discovery-insights-portfolio/analysis-and-visualization/qiagen-clc-genomics-workbench/
TFT:TFT legacy	GSEA	gsea-msigdb.org
WormCat (version 2)	Wormcat	http://www.wormcat.com/
CeNGEN	CeNGEN	CeNGEN
TFBIND	TFBIND	tfbind.hgc.jp.
WormEnrichr	WormEnrichr	maayanlab.cloud/WormEnrichr
Prism (version 10.1.0)	GraphPad	https://www.graphpad.com/

**Other**		

Gibson Assembly	NEB	Cat#E2611L
Q5 high fidelity polymerase	NEB	Cat#M0491S
Monarch PCR and DNA cleanup kit	NEB	Cat#T1030S
Precellys tubes	Thermo Fisher Scientific	Cat#02-682-556
Precellys Evolution homogenizer	Bertin Instruments	Cat#P000062-PEVO0-A.0

### Resource availability

#### Lead contact

Further information and requests for resources and reagents should be directed to and will be fulfilled by the lead contact, Peter M. Douglas (peter.douglas@utsouthwestern.edu).

#### Materials availability

All *C. elegans* strains made for this study will be available upon request. Strain details are provided below and in the key resources table.

#### Data and code availability

All transcriptomic datasets generated during this study are publicly accessible and are deposited in the NCBI Gene Expression Omnibus under the accession numbers GSE254142 (combined), GSE254138 (PMD216 on EV, *cat-1*, and *cat-2* RNAi), and GSE254141 (N2 injury timeline).

This study does not report any original code.

Any additional information required to reanalyze the data reported in this paper is available from the lead contact upon request.

### Method details

#### *C. elegans* strains and maintenance

All strains were maintained on standard NGM plates seeded with OP50 bacterial lawn and were kept in a dark 15°C incubator. For experiments requiring large numbers of worms, stock plates were chunked onto OP50 seeded HGM plates and incubated at 20°C and age-synchronization was performed by hypochlorite treatment of gravid animals.

The following strains were obtained from the CGC: N2, CF512 *(rrf-3(b26) II; fem-1(hc17) IV.)*, WM27 (*rde-1(ne219) V*), XE1474 (*dat-1p::rde-1::SL2::sid-1 + unc-119(+); wpSi6 II; eri-1(mg366) IV; rde-1(ne219) V; lin-15B(n744) X*). TQ296 (*trp-4(sy695) I*).

The following strains were made in our laboratory previously^14^ and used in this study:

PMD13 (*rrf-3(b26) II; fem-1(hc17) IV; egIs1 [dat-1p::GFP]*).

PMD14 (*rrf-3(b26) II; fem-1(hc17) IV; juIs76 [unc-25p::GFP* + *lin-15(+)] II*).

PMD25 (*mgIs42 [tph-1::GFP + rol-6(su1006)]; CF512 [fer-15(b26) II; fem-1(hc17ts) IV]*).

PMD63 (*rrf-3(b26) II; fem-1(hc17) IV; egIs1 [dat-1p::GFP]; sid-1(pk3321) V; uIs69 [pCFJ90 (myo-2p::mCherry) + unc-119p::sid-1]*).

The following strains were made for this study:

PMD184 (utsEx18 *[rgef-1p::ORP1::roGFP2::T2A::mCherry::unc-54 3’ UTR]*).

PMD185 (utsEx19 *[dat-1p::ORP1::roGFP2::T2A::mCherry::unc-54 3’ UTR]*).

PMD188 (utsEx22 *[tph-1p::ORP1::roGFP2::T2A::mCherry::unc-54 3’ UTR]*).

PMD189 (utsEx23 *[dat-1p::MTS::ORP1::roGFP2::T2A::mCherry::unc-54 3’ UTR]*).

PMD227 (utsEx44 *[dat-1p::55aaTOMM20::ORP1::roGFP2::T2A::mCherry::unc-54 3’ UTR])*.

PMD249 (utsEx19 *[dat-1p::ORP1::roGFP2::T2A::mCherry::unc-54 3’ UTR]; wpSi6 [dat-1p::rde-1::SL2::sid-1 + unc-119(+)] II; eri-1(mg366) IV; rde-1(ne219) V; lin-15B(n744) X.)* was made by crossing PMD185 (utsEx19 *[dat-1p::ORP1::roGFP2::T2A::mCherry::unc-54 3’ UTR]*) with XE1474 (*dat-1p::rde-1::SL2::sid-1 + unc-119(+); wpSi6 II; eri-1(mg366) IV; rde-1(ne219) V; lin-15B(n744) X*).

PMD238 (utsEx16 *[unc-25p::CAT-2::mCherry::unc-54 3’ UTR; unc-25p::BAS-1::mCherry::unc-54 3’ UTR]*) and was crossed with PMD14 (*rrf-3(b26) II; fem-1(hc17) IV; juIs76 [unc-25p::GFP* + *lin-15(+)] II*) to make PMD146 (utsEx16 *[unc-25p::CAT-2::mCherry::unc-54 3’ UTR; unc-25p::BAS-1::mCherry::unc-54 3’ UTR]; rrf-3(b26) II; fem-1(hc17) IV; juIs76 [unc-25p::GFP* + *lin-15(+)] II)]*).

PMD239 (utsEx17 *[tph-1p::CAT-2::mCherry::unc-54 3’ UTR; tph-1p::BAS-1::mCherry::unc-54 3’ UTR]*) and was crossed with PMD25 (*mgIs42 [tph-1::GFP + rol-6(su1006)]; CF512 [rrf-3(b26) II; fem-1(hc17ts) IV]*) to make PMD147 (utsEx17 *[tph-1p::CAT-2::mCherry::unc-54 3’ UTR; tph-1p::BAS-1::mCherry::unc-54 3’ UTR]; mgIs42 [tph-1::GFP + rol-6(su1006)] rrf-3(b26) II; fem-1(hc17ts) IV*).

PMD216 (*fem-1(hc17) IV, rrf-3(b26) II; egIs1 [dat-1p::GFP]; dat-1p::rde-1::SL2::sid-1 + unc-119(+); wpSi6 II; eri-1(mg366) IV; rde-1(ne219) V; lin-15B(n744) X*) was made by crossing PMD13 (*rrf-3(b26) II; egIs1 [dat-1p::GFP]*) with XE1474 (*dat-1p::rde-1::SL2::sid-1 + unc-119(+); wpSi6 II; eri-1(mg366) IV; rde-1(ne219) V; lin-15B(n744) X*).

PMD191 (*fem-1(hc17) IV, rrf-3(b26) II; egIs1 [dat-1p::GFP], trp-4(sy695) I*) was made by crossing PMD13 (*rrf-3(b26) II; egIs1 [dat-1p::GFP]*) and TQ296 (*trp-4(sy695) I*).

PMD190 (*fem-1(hc17) IV, rrf-3(b26) II; egIs1 [dat-1p::GFP]*) was made by crossing PMD13 (*rrf-3(b26) II; egIs1 [dat-1p::GFP]*) and TQ296 (*trp-4(sy695) I*) but selected for trp-4 WT allele to use as a control for PMD191.

PMD156 (*fem-1(hc17) IV, rrf-3(b26) II; egIs1 [dat-1p::GFP], cat-2(n4547) II*) was made by crossing PMD13 with MT15620 (*cat-2(n4547) II.*).

RDE-1 and SID-1 mutants were always confirmed by feeding of *cdc-25.1* RNAi which creates sterile animals when there is functional RDE-1 in the germline. Transgenic rescue of RDE-1 or SID-1 expression in the tissue of interest was confirmed using GFP RNAi.

#### Molecular cloning

All cloning was done using Gibson assembly (NEB E2611L) following the standard NEB cloning protocol. Briefly, all inserts were amplified including a 15 to 20 nucleotide overhang with homology to the recombination targets. All cloning PCR reactions were done with the Q5 high fidelity polymerase (NEB M0491S). Amplified PCR products were run on an agarose gel to confirm clean amplification and 3 x 50 μl PCR reactions were combined and purified using the Monarch® PCR & DNA Cleanup Kit (NEB T1030S). Insert and vector were mixed at the recommended 3-fold molar excess of the insert and at least 100 ng of PCR linearized vector backbone. Gibson assembly master mix (2x) was then added to the mixture and incubated at 50°C for 15 minutes and was then used to transform NEB 5-alpha competent *E. coli* cells. All promoter regions as well as the *bas-1* and *cat-2* genes used in this study were amplified from genomic *C. elegans* DNA and were cloned into pNE1^14^ backbone containing a 3’ GSGGSG linker, mCherry, and unc-54 3’ UTR. ORP1-roGFP2 was cloned from transgenic *Drosophila Melanogaster* (provided by Dr. Matthew Sieber), inserted into pNE1, and the GSGGSG linker was replaced by a T2A self-cleaving peptide sequence (dsDNA oligo synthesized by IDT). All primers used are listed in SI Table S1.

#### Worm microinjections

Microinjections were performed as described previously.^14^ All injected plasmids were PCR linearized using Forward primers targeting the 5’ start of the promoter region and Reverse primers targeting the 3’ end of the *unc-54* 3’UTR to increase expression in neurons.^37^ PCR products were then purified using PCR purification kit (NEB T1030S) and DNA was diluted in molecular grade water to a final concentration of 100 ng/μl and loaded into a pulled glass capillary (with an inner diameter of 0.78 mm and outer diameter of 1.0 mm OD) after which the tip of the capillary was carefully broken against the edge of a glass coverslip. Worms then were injected using a FemtoJet 4i (Eppendorf, Hamburg, Germany) attached to a Leica DMi8 inverted stereomicroscope using pulled glass capillaries. Young N2 worms were placed on an agar padded coverslip in a drop of Halocarbon 700 oil (Sigma Aldrich H8898) and were injected in each arm of the gonad. After injection, worms were recovered on OP50 seeded plates and progeny was monitored for transgene expression.

#### Single worm genotyping

Single worms were picked using a worm pick and transferred to a tube containing 10 μl of worm genotyping lysis buffer (10 mM Tris pH8.2, 50 mM KCl, 2.5 mM MgCl_2,_ 0.45% Tween, 60 μg/ml proteinase K). Tubes were frozen for 15 minutes at -80°C and subsequently lysed at 60°C for 60 minutes, and 95°C for 15 minutes. 5 μl of the lysate was then used in a standard PCR reaction which was analyzed on a 1% agarose gel. All primers used are listed in SI Table S1.

#### Formaldehyde induced fluorescence and confocal microscopy

Ectopic dopamine in serotonergic neurons was visualized using formaldehyde induced fluorescence (FIF) using the simplified and adapted protocol found here.^23^^,^^38^ Briefly, 10 to 20 L3-L4 larvae were placed on a glass slide in a drop of 4% formaldehyde in 0.1 M Na2HPO4/KH2PO4 buffer, pH 7.2. After 5 minutes incubation at RT excess liquid was removed using 3MM filter paper and the slide was placed on a heat block set at 96°C for 10 minutes. During this step the slide was covered with aluminum foil to trap heat and formaldehyde vapors. Once the slide cooled to RT a drop of glycerol was placed on top of the worms and a coverslip was applied. Fluorescence was visualized using 405nm excitation and 470nm – 700nm emission at 63x magnification. Micrographs were acquired using a Leica SP8 confocal microscope and Leica Application Suite X (LAS X) software (version 3.5.5). All images were acquired in xyz acquisition mode, and each line was imaged three times and averaged to reduce noise. Z-stack acquisitions were then used to render 3D projections using LAS X’ topological 3D visualization tool. Laser power, range and gain remained consistent between trials.

Dopaminergic ROS sensor strains were imaged on glass slides and immobilized by levamisole dissolved in M9 buffer. Micrographs were acquired using a Leica SP8 confocal microscope and Leica Application Suite X (LAS X) software (version 3.5.5). All images were acquired in xyz acquisition mode. Fluorescent Z-stack acquisitions are shown as maximum intensity using the ImageJ Z-project function. For DIC Z-stacks a single representative slice is shown. Laser power, range and gain remained consistent between strains.

#### *C. elegans* blunt force trauma

Trauma was administered to *C. elegans* using our blunt force trauma model of mechanical stress described here.^14^ Briefly, age-synchronized worms were grown on empty vector control (EV) or the respective RNAi. Temperature-restrictive strains were grown at 25°C to avoid generation of progeny. Alternatively, strains lacking temperature sensitive mutations were grown on *cdc-25.1* RNAi to induce sterilization yet were still grown at 25°C to allow for comparison with temperature sterile strains. On day 1 of adulthood, worms were rinsed off growth plates in liquid M9 buffer and pelleted by centrifugation at 1000 x g for 30 seconds. 125 μl worm pellets were then transferred to 2 ml Precellys tubes (Thermo Fisher Scientific 02-682-556) and M9 was added to a total volume of 500 μl. Worms were then subjected to high-frequency, multidirectional agitation in a Precellys Evolution homogenizer (Bertin Instruments P000062-PEVO0-A.0) for 16 s at 8600 rpm. Worms were pelleted at 1000 x g for 30 seconds and transferred to fresh recovery plates containing the respective RNAi or EV on which they were grown prior to trauma. Non-injured control worms were suspended in M9 for comparable lengths of time, subjected to the same number of washes and centrifugation steps, and then transferred to recovery plates without having received trauma.

#### RNAi administration

Worm strains were grown on HT115 *E. coli* expressing RNAi constructs from either the Vidal^39^ or Ahringer RNAi libraries.^40^ The L4440 empty vector control (EV) RNAi construct was used for control treatments. RNAi strains were grown in small starter cultures for 8 hours prior to inoculating larger cultures in Terrific Broth (TB) containing carbenicillin, and grown for 15 hours on an orbital shaker at 37°C. After 15 hours, cultures were induced with 1 mM IPTG and incubated for an additional 4 hours at 37°C to ensure RNAi expression. Cultures were then centrifuged at 4000 x g for 10 minutes and bacterial pellets were re-suspended in 1/5^th^ of the original culture volume in TB before being spread on 100 mm NGM plates containing a final concentration of 1 mM IPTG and 0.1 mg/ml carbenicillin. When combining multiple RNAi constructs on one plate, bacterial cultures were mixed at equal volumes prior to plate seeding. All RNAi constructs used are listed in SI Table S2.

#### Large particle flow Cytometry collection and data analysis

Worms were analyzed using the COPAS FP-250 large particle flow cytometer (Union Biometrica) in combination with the LP Sampler (Union Biometrica) to automate sample acquisition. Worms were washed off 100 mm plates using M9 and further washed 3 times with M9 before transferring a 100 μl worm pellet into a 96-well plate for acquisition. All samples were always spaced by wells containing M9 only to avoid cross well collection. COPAS GP SHEATH REAGENT (Union Biometrica 300-5070-100) was used as a flow sheath solution. Extinction was measured using the 488 nm laser and a 1.3 neutral density filter for all experiments except for ROS sensor strains (PMD184, PMD185, PMD188, PMD189, and PMD227) which were acquired using the 561 nm laser and a 2.0 neutral density filter to allow for 405 nm measurements without any 488 nm excitation. Laser power and PMT voltage was optimized empirically for each individual strain and was subsequently kept consistent for each experiment. The gain setting was always set at 1.0 for extinction and 2.0 for all fluorescent channels. Data was collected and processed using Union Biometrica FlowPilot software (version 1.6.1.8) and further normalization was performed in Excel and statistical analysis and outlier identification was done in GraphPad Prism (version 10.1.0).

#### Oxidation ratio

To be able to compare ROS formation across conditions we created an oxidation ratio described below. Since all ROS sensor strains were maintained as extrachromosomal transgenic worm strains, we were able to account for background fluorescence by looking at the mCherry negative (mCh-) population and subtracting the mean GFP fluorescence per condition from the mCherry positive (mCh+) population. This background normalized 405/488 ratio from the injured population was then normalized to time-matched, uninjured controls via subtraction of the average uninjured, blank normalized, 405/488 ratio.

$$Oxidation\;ratio=Injured\frac{\left(405\;{mCh}^{+}\;-\;mean\;405\;{mCh}^{-}\right)}{\left(mean\;488\;{mCh}^{+}\;-mean\;488\;{mCh}^{-}\right)}\;—uninjured\frac{\left(mean\;405\;{mCh}^{+}\;-\;mean\;405\;{mCh}^{-}\right)}{\left(mean\;488\;{mCh}^{+}\;-mean\;488\;{mCh}^{-}\right)}$$

#### Dopaminergic GFP index

To compare the delta dopaminergic GFP retention between EV and RNAi conditions, peak height GFP was normalized as described previously.^14^ Briefly, the GFP retention of individual injured worms was calculated by dividing the GFP peak height value by the average GFP peak height value of uninjured controls from the corresponding genotype or RNAi condition. To calculate the Dopaminergic GFP index, which allows for cross-trail comparison between RNAi treated or mutant worms, GFP retention percentages were normalized to EV or WT controls using the formula below. As a result, any value >0 can be interpreted as protective while any value <0 can be interpreted as degenerative compared to control conditions. Neuronal GFP quantification was always obtained from head neurons exclusively. This was achieved by applying a partial profile of 20% of the head region using the Union Biometrica FlowPilot software (version 1.6.1.8). Orientation of the worm was always established by ‘highest peak height GFP signal in the head’.

$$Dopaminergic\;GFP\;index=\frac{\left(control\;GFP\;retention\Delta -test\;GFP\;retention\Delta \right)}{\left(control\;GFP\;retention\Delta -1\right)}$$

#### RNA extraction

RNA was extracted from 1500-2000 worms per condition using phenol-chloroform extraction. Briefly, 1 ml of Trizol was added to 100 μL of age synchronized worm pellets and then flash-frozen in liquid nitrogen. Worms were then lysed by repeated freeze-thaw cycles and 200 μL of chloroform was added and incubated for 3 min at RT. The aqueous phase was separated by spinning at 12000 x g for 10 minutes at 4°C and transferred to a new tube for isopropanol/ethanol precipitation of RNA.

#### RNA-sequencing

Quality control, mRNA purification, and paired-end 150 bp Illumina sequencing were performed by Novogene (Sacramento, CA). mRNA was enriched using oligo(dT) beads, randomly fragmented in fragmentation buffer, and reverse transcribed to cDNA using random hexamers. Following first-strand synthesis, Illumina synthesis buffer was added with dNTPs, RNase (H) and *E. coli* polymerase I to synthesize the second strand by nick-translation. The cDNA library was purified, underwent terminal repair, A-tailing, and ligation of adapters before PCR enrichment. The cDNA library concentration was quantified with a Qubit 2.0 fluorometer (ThermoFisher Scientific) and sized with an Agilent 2100 Bioanalyzer (Agilent). RNA-seq statistical analysis was performed using CLC software (version 9.0, CLC Bio, Aarhus, Denmark). Data is presented as reads per kilobase million (RPKM) with values normalized to control levels made relative to 0.

#### Quantitative PCR

1 μg of extracted RNA was reverse transcribed using a QuantiTect Reverse Transcription kit (Qiagen 205311) according to manufacturer’s protocol, which includes genomic DNA wipeout. qPCR was performed using the iTaq Universal SYBR Green Supermix kit (Bio-Rad 1525125) in combination with the CFX384 Real-Time System (Bio-Rad). 50 ng of cDNA and a primer concentration of 300 nM per primer was used for each well. All samples were loaded in technical triplicate and biological replicates of 3 or more. Selectivity of primer pair amplification products were determined by melting curve analysis and wells with more than 1 melt peak were excluded from analysis. The relative transcript abundance was calculated through normalization to housekeeping genes *tba-1* and *Y45F10D.4* using the ΔΔCt method.

#### Bioinformatic screening and identification of transcription factor candidates

TFT:TFT legacy analysis was performed to identify transcription factors with differential expression of downstream targets using gsea-msigdb.org and the top 50 hits were used for further analysis. Other filtering constraints included that transcription factors must have annotated homologues in both mice and worm, must be annotated to be expressed in dopaminergic neurons according to CeNGEN, and have predicted binding sites in the promoter region (4 kb upstream of transcription start site) of human *TH* according to tfbind.hgc.jp. Gene set enrichment analysis of *cat-1* RNAi responsive, and *cat-2* RNAi resistant DEGs was performed using WormEnrichr (maayanlab.cloud/WormEnrichr)^41^^,^^42^

### Quantification and statistical analysis

All statistical analyses were conducted using GraphPad Prism (version 10.1.0) and Qiagen Bioinformatics CLC Genomics Workbench (version 9.5). For large worm populations, outliers were removed using the ROUT method (Q = 1%) prior to analysis. All ANOVA F-statistics are reported in SI Table S3.

## Supplemental information

Document S1. Figures S1‒S5 and Tables S1‒S3
