# Supplementary material for: Cytosolic dopamine determines hypersensitivity to blunt force trauma
Source: iScience. 2024 May 23;27(6):110094. doi: 10.1016/j.isci.2024.110094 (PMC11179581; doi:10.1016/j.isci.2024.110094)
Supplement: Document S1. Figures S1‒S5 and Tables S1‒S3 [file mmc1.pdf]

iScience, Volume *27*

Supplemental information

Cytosolic dopamine determines hypersensitivity to blunt force trauma

Kielen R. Zuurbier, Rene Solano Fonseca, Sonja L.B. Arneaud, Lexus Tatge, Gupse Otuzoglu, Jordan M. Wall, and Peter M. Douglas

Supplemetal Information (SI)

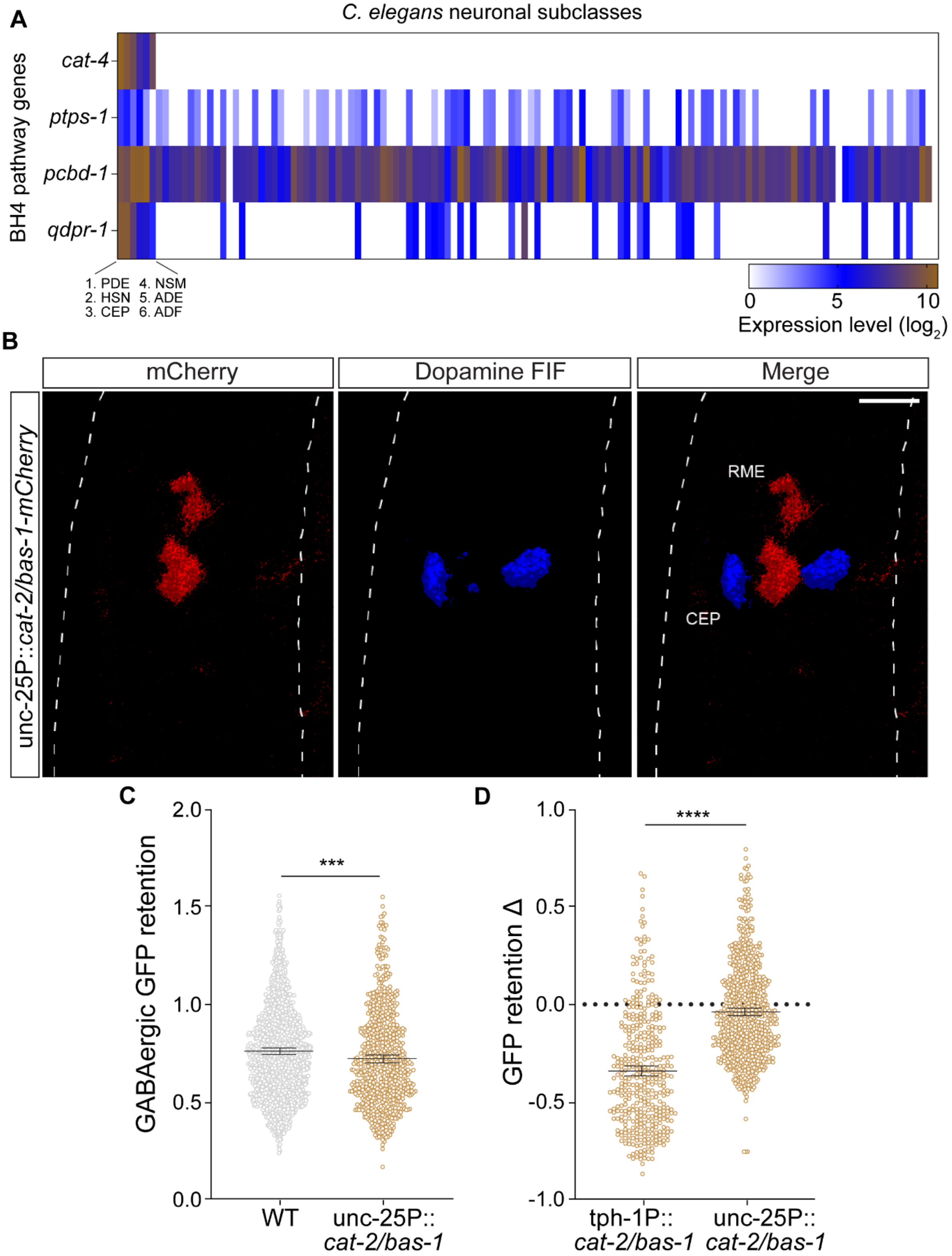

Figure S1. Expression of dopaminergic enzymes in GABAergic neurons does not drive dopamine synthesis. Related to Figure 1.

(A) CeNGEN expression levels of BH4 synthesis (*cat-4* and *ptps*-1) and recycling (*pcbd-1* and *qdpr-1*) in all *C. elegans* neurons. Only dopaminergic and serotonergic neurons express all 4 genes.

(B) Fluorescence micrograph shows the three-dimensional renders of worm head regions compiled from z-stack images. Fixed worms are stained for dopamine (blue) using formaldehyde-induced fluorescence (FIF) and compared with mCherry (red) expression. CEP annotation indicates the cephalic dopamine neurons, RME indicates GABAergic neuron in the *C. elegans* head region. White dashed line indicates the worm outline. Scale bar = 5 μM.

(C) GABAergic GFP retention in non-transgenic controls versus the transgenic worms (PMD146) ectopically expressing dopamine (*unc-25P*::*cat-2/bas-1::mCherry*) 48 hours after injury. Each point represents an injured worm normalized to the mean of the uninjured control population. Plots show mean ± 95%CI. WT $n = 1857$ and *tph-1P*::*cat-2/bas-1::mCherry* $n = 776$. Statistical analysis was performed using an unpaired t-test; $p = <0.0007$.

(D) GFP retention of serotonergic (PMD147) vs GABAergic (PMD146) mCherry+ populations normalized to uninjured mCherry+ control. Statistical analysis was performed using an unpaired t-test; $p = <0.0001$. *tph-1P*::*cat-2/bas-1* $n = 412$ and *unc-25P*::*cat-2/bas-1* $n = 776$.

*= $p < 0.05$, **= $p < 0.01$, ***= $p < 0.001$, and ****= $p < 0.0001$

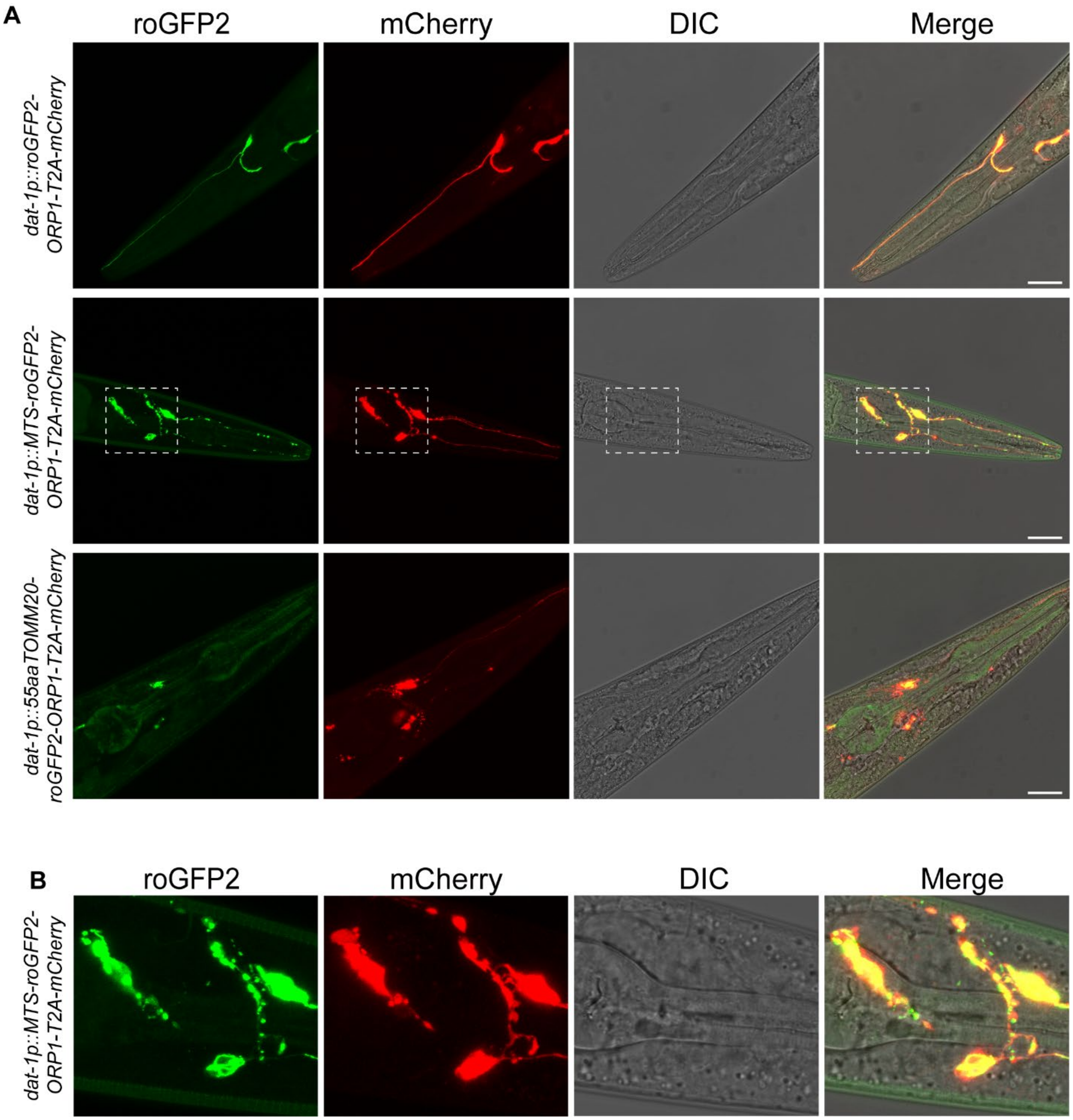
A
roGFP2
mCherry
DIC
Merge
dat-1p::roGFP2-ORP1-T2A-mCherry
dat-1p::MTS-roGFP2-ORP1-T2A-mCherry
dat-1p::55aaTOMM20-roGFP2-ORP1-T2A-mCherry
B
roGFP2
mCherry
DIC
Merge
dat-1p::MTS-roGFP2-ORP1-T2A-mCherry

Figure S2. Validation of dopaminergic expression of genetically encoded ROS sensors. Related to Figure 2.

(A) Fluorescence micrographs and differential interference contrast (DIC) images of Dopaminergic ROS sensor strains (top) PMD185 (utsEx19 *[dat-1p::ORP1::roGFP2::T2A::mCherry::unc-54 3'UTR]*), (middle) PMD189 (utsEx23 *[dat-1p::MTS::ORP1::roGFP2::T2A::mCherry::unc-54 3'UTR]*) , (bottom) PMD227 (utsEx44 *[dat-1p::55aaTOMM20::ORP1::roGFP2::T2A::mCherry::unc-54 3'UTR]*). Scale bars = 20 µM. Dashed boxes indicate regions blown up and shown in **B**. roGFP2 images only show 488nm excited signal (reduced state).

(B) Zoomed in images from PMD189 (utsEx23 *[dat-1p::MTS::ORP1::roGFP2::T2A::mCherry::unc-54 3'UTR]*) shown in **A**.

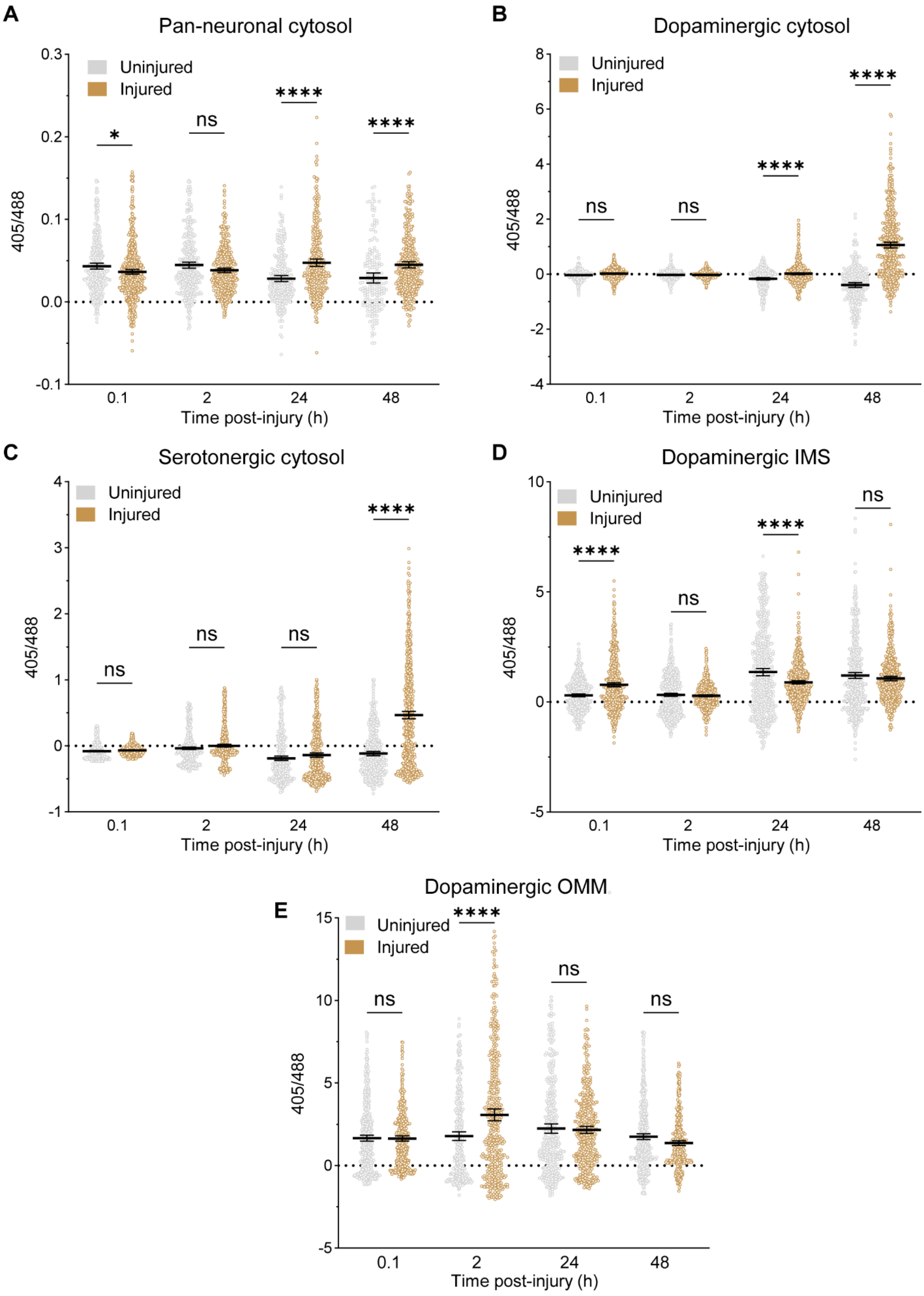
A
Pan-neuronal cytosol
Uninjured
Injured
405/488
Time post-injury (h)
B
Dopaminergic cytosol
C
Serotonergic cytosol
D
Dopaminergic IMS
E
Dopaminergic OMM
ns
****
*

Figure S3. Raw 405/488 ROS sensors ratios in injured and uninjured *C. elegans*. Related to Figure 2

(A) Cytosolic ROS production in all head neurons after injury of roGFP2-ORP1 expressing transgenic worms (PMD184 utsEx18 *[rgef-1p::ORP1::roGFP2::T2A::mCherry::unc-54 3' UTR]*). 405nm/488nm oxidation ratio is calculated after normalization to the background fluorescence of non-transgenic controls. Statistical analysis was performed using an ordinary one-way ANOVA Tukey corrected multiple comparisons. Grey = uninjured, gold = injured. Mean ± 95%CI. From left to right, n = 342, 570, 363, 440, 284, 336, 178, and 347 collected over 3 individual experiments.
(B) Cytosolic ROS production in dopaminergic neurons in injured and uninjured roGFP2-ORP1 expressing transgenic worms (PMD185 utsEx19 *[dat-1p::ORP1::roGFP2::T2A::mCherry::unc-54 3' UTR]*). 405nm/488nm oxidation ratio is calculated after normalization to the background fluorescence of non-transgenic controls. Statistical analysis was performed using an ordinary one-way ANOVA with Šidák's multiple comparisons test. Grey = uninjured, gold = injured. Mean ± 95%CI. From left to right, n = 698, 796, 548, 642, 350, 526, 260, and 552 collected over 5 individual experiments.
(C) Cytosolic ROS production in serotonergic neurons in injured and uninjured roGFP2-ORP1 expressing transgenic worms (PMD188 utsEx22 *[tph-1p::ORP1::roGFP2::T2A::mCherry::unc-54 3' UTR]*). 405nm/488nm oxidation ratio is calculated after normalization to the background fluorescence of non-transgenic controls. Statistical analysis was performed using an ordinary one-way ANOVA with Šidák's multiple comparisons test. Grey = uninjured, gold = injured. Mean ± 95%CI. From left to right, n = 345, 493, 326, 454, 102, 150, 122, and 205 collected over 3 individual experiments.

(D) Inner mitochondrial ROS production in dopaminergic neurons in injured and uninjured roGFP2-ORP1 expressing transgenic worms (PMD189 utsEx23 *[dat-1p::MTS::ORP1::roGFP2::T2A::mCherry::unc-54 3' UTR]*). 405nm/488nm oxidation ratio is calculated after normalization to the background fluorescence of non-transgenic controls. Statistical analysis was performed using an ordinary one-way ANOVA with Šidák's multiple comparisons test. Grey = uninjured, gold = injured. Mean ± 95%CI. From left to right, n = 554, 633, 657, 573, 524, 641, 600, and 483 collected over 4 individual experiments.

(E) Outer mitochondrial ROS production in dopaminergic neurons in injured and uninjured roGFP2-ORP1 expressing transgenic worms (PMD227 utsEx44 *[dat-1p::55aaTOMM20::ORP1::roGFP2::T2A::mCherry::unc-54 3' UTR]*). 405nm/488nm oxidation ratio is calculated after normalization to the background fluorescence of non-transgenic controls. Statistical analysis was performed using an ordinary one-way ANOVA with Šidák's multiple comparisons test. Grey = uninjured, gold = injured. Mean ± 95%CI. From left to right, n = 483, 450, 376, 433, 403, 434, 530, and 446 collected over 3 individual experiments.

*= $p < 0.05$, **= $p < 0.01$, ***= $p < 0.001$, and ****= $p < 0.0001$

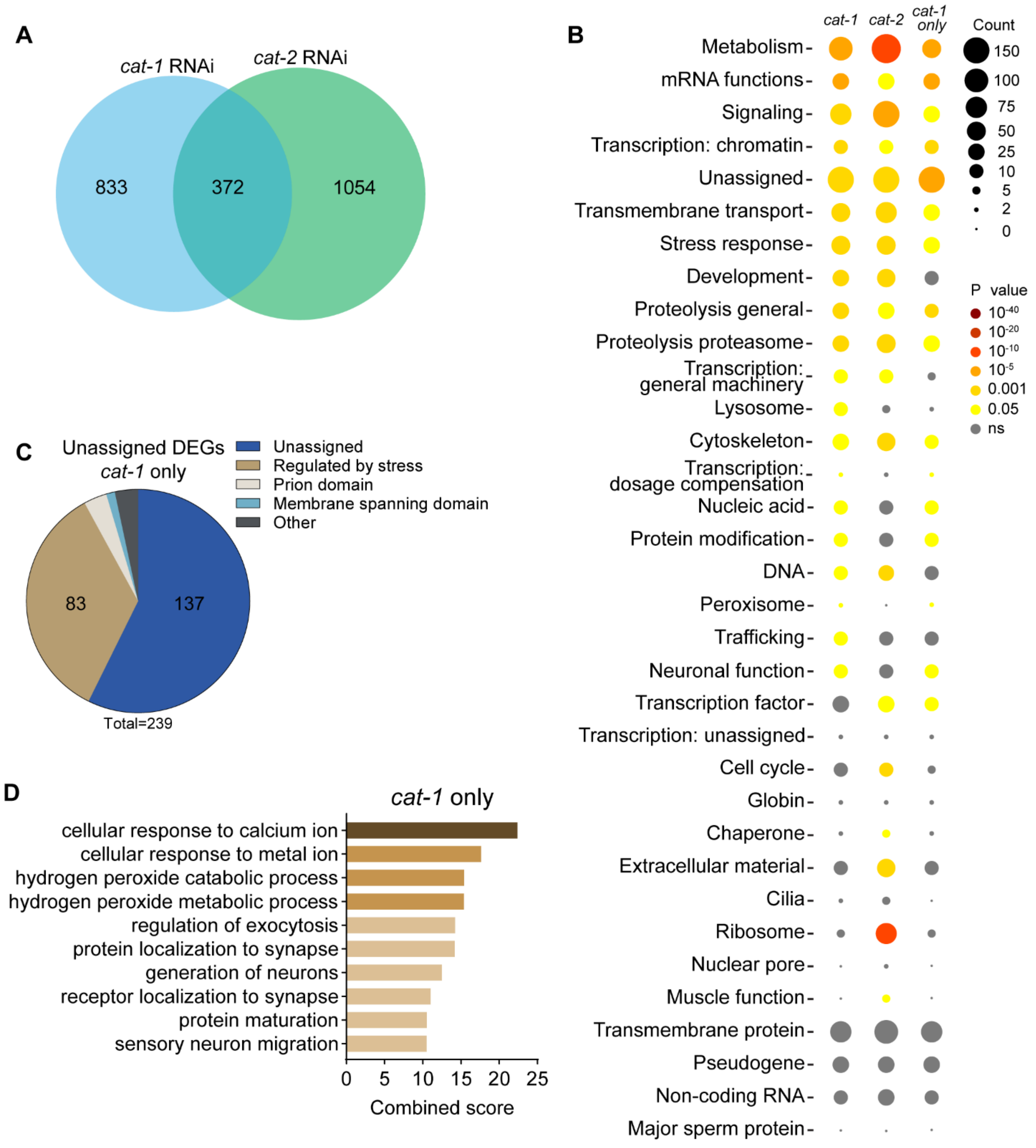

A
cat-1 RNAi
cat-2 RNAi
833
372
1054
B
cat-1
cat-2
cat-1 only
Count
150
100
75
50
25
10
5
2
0
P value
10-40
10-20
10-10
10-5
0.001
0.05
ns
Metabolism
mRNA functions
Signaling
Transcription: chromatin
Unassigned
Transmembrane transport
Stress response
Development
Proteolysis general
Proteolysis proteasome
Transcription: general machinery
Lysosome
Cytoskeleton
Transcription: dosage compensation
Nucleic acid
Protein modification
DNA
Peroxisome
Trafficking
Neuronal function
Transcription factor
Transcription: unassigned
Cell cycle
Globin
Chaperone
Extracellular material
Cilia
Ribosome
Nuclear pore
Muscle function
Transmembrane protein
Pseudogene
Non-coding RNA
Major sperm protein
C
Unassigned DEGs
cat-1 only
Unassigned
Regulated by stress
Prion domain
Membrane spanning domain
Other
83
137
Total=239
D
cat-1 only
cellular response to calcium ion
cellular response to metal ion
hydrogen peroxide catabolic process
hydrogen peroxide metabolic process
regulation of exocytosis
protein localization to synapse
generation of neurons
receptor localization to synapse
protein maturation
sensory neuron migration
0
5
10
15
20
25
Combined score

Figure S4. Impaired vesicular packaging of dopamine activates stress response pathways. Related to Figure 4

(A) Venn diagram depicting overlap of differentially expressed genes ($p < 0.05$) identified through RNA-seq in PMD216 day 1 adults raised on *cat-1/SLC18A2* or *cat-2/TH* RNAi compared to EV control. $n = 4$ samples per condition

(B) WormCat (category 1) gene set enrichment analysis of differentially expressed genes (DEG) identified in **A**. Dot size corresponds to the number of genes associated with the gene set while color indicates significance.

(C) Piechart depicting WormCat category 3 annotation of all category 1 'unassigned' cluster in **B**.

(D) Gene set enrichment analysis of 833 genes differentially expressed upon *cat-1* RNAi yet unchanged upon *cat-2* RNAi. GeneRIF GO Biological Process was used as annotation source.

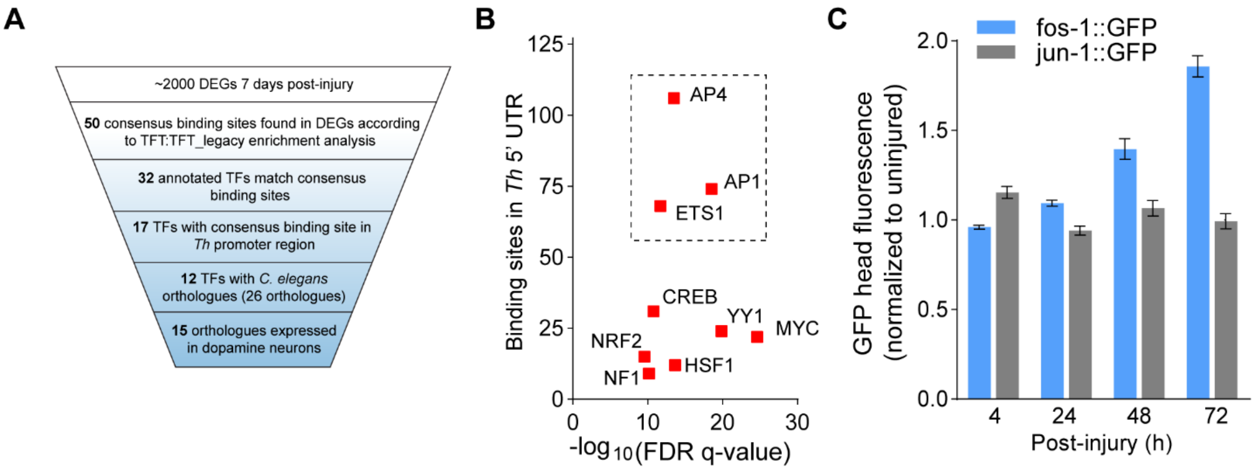

Figure S5. Candidate-based screening strategy to identify trauma induced activators of dopamine synthesis. Related to Figure 5.

(A) Filtering strategy for TF identification of *cat-2* induction.

(B) Predicted binding sites in the promoter of mouse tyrosine hydroxylase of TFs that passed all criteria in **A** vs their TFT:TFT gene set enrichment analysis following brain injury.

(C) GFP quantification of injured worms expressing FOS-1 or JUN-1 fused to GFP. All data is normalized to ag-matched uninjured control. Mean ± 95%CI. From left to right FOS-1 *n*= 2097, 4457, 2009, and 2226. JUN-1 *n*= 782, 1439, 777, and 809. All samples were collected over 3 individual replicates.

Supplementary Tables

Table S1. Primers used in this study. Related to STAR methods.

Primer Name	Primer Sequence (5'-3')
**qPCR primers**	
dat-1 qPCR Fw	CGCCTCATTTATTCCATTGGT
dat-1 qPCR Rv	ACTCGTTGCCATTTCTCAG
cat-2 qPCR Fw 5-6	GACGGTTTATTGGTTCATTGTGG
cat-2 qPCR Rv 5-6	AATTCTCCATACGCCGACAG
**Genotyping primers**	
CAT-2 geno Fw1	GTGTCCGGAAAATAGTATCGC
CAT-2 geno Fw2	CATGGTAACTTAGAATTAACACCC
CAT-2 geno Rv	TTAGTAGCCTTGCACGCTAG
trp-4 geno Fw1	TTCCTCCTGAAACAGAAGCATGACAC
trp-4 geno Fw2	CTACCAGCCTGTCGACGAGGAC
trp-4 geno Rv	TGGTTCGCCCGTCTGTAAGCT
**Primers to clone roGFP-ORP1 under control of dat-1, tph-1, or rgef-1 promoters**	
BB-DAT-3'-Rv	GGCTAAAAATTGTTGAGATTCGAGTAAACCGTA
roGFP-5'-Fw	GTGAGCAAGGGCGAGGAGCTGTTCA
roGFP2-Orp1 Fw	ATGGTGAGCAAGGGCGA
roGFP2-orp1 Rv	CTAGACTATTCCACCTCTTTCAAAAGTT
BB-DAT-ORP Rv	GCCCTTGCTCACCATGGCTAAAAATTGTTGAGATTCGAG
BB-TPH-ORP Rv	GCCCTTGCTCACCATCGAATCCATATGATTGAAGAGAGCA
BB-RGEF-ORP Rv	CGCCCTTGCTCACCATGATCCTTTACTGCTGATCGTC
ORP-2A Fw	ATGGTGAGCAAGGGCGA
ORP-2A Rv	GGATCCACGTCCCTCTTCCACCTCTTTCAAAAGTTCTT
BB-X-X Fw	GGAGAACCCAGGACCAGTGAGCAAGGGCGAGGA
**Primers to clone CAT-2 and BAS-1 under control of tph-1 or unc-25 promoters**	
CAT-2 cloning Fw1	ATGTTTTTGCGGTATATGCGGAGG
CAT-2 cloning Rv1	TCACATTGTAATCGATATTTTCATCCGATCAGC
BAS-1 cloning Fw1	ATGGACTCCCAAAAATTGCGGACT
BAS-1 cloning Rv1	TTAGAATAATTTCTCGGCGAGTTCAAAGATGACG
BAS-1 cloning Rv2	GAATAATTTCTCGGCGAGTTCAAAGATGACG
CAT-2 cloning Rv2	CATTGTAATCGATATTTTCATCCGATCAGC
BB+BAS-1 Fw1	GCCGAGAAATTATTCGGATCCGGAGGATCCGGAGT
BB+CAT-2 Fw1	ATGAAAATATCGATTACAATGGATCCGGAGGATCCGGAG
BB Rv1	AGCTTATTTCATTTCCAAGTTGTTAGCGTATCCATCGTTGTGAGTG
TPH-1 BB Fw1	TTGGAAATGAAATAAGCTTAAGTTATTGTACCCTGACCAA
TPH-1 BAS-1 Rv1	CAATTTTTGGGAGTCCATCGAATCCATATGATTGAAGAGAG
TPH-1 CAT-2 Rv1	GCATATACCGCAAAAACATCGAATCCATATGATTGAAGAGAG
UNC-25 BB Fw1	ACTTGGAAATGAAATAAGCTCCGGAAATTCGAAATTTTTAAC
UNC-25 BAS-1 Rv1	AATTTTTGGGAGTCCATTTTTGGCGGTGAACTGA
UNC-25 CAT-2 Rv1	ATATACCGCAAAAACATTTTTGGCGGTGAACTGA

BB+BAS-1 Fw2	GCCGAGAAATTATTCGG
BB+CAT-2 Fw2	ATGAAAATATCGATTACAATGG
BB Rv2	AGCTTATTTCATTTCCAAGT
**Linearizing primers for worm microinjections**	
dat-1p lin inj Fw	CCATGAAATGGAACTTGAATCC
unc-25p lin inj Fw	CCGGAAATTCGAAATTTTTAACG
tph-1p lin inj Fw	TAAGTTATTGTACCCTGACCAAAAC
unc-54 lin inj Rv	AAACAGTTATGTTTGGTATATTGGG

Table S2. RNAi constructs used in this study. Related to STAR methods.

Gene target	Sequence name	Library
EV	L4440	N/A
cat-1	W01C8.6	Vidal
cat-2	B0432.5	Vidal
dop-2	K09G1.4	Ahringer
mcu-1	K02B2.3	Ahringer
kin-1	ZK909.2	Vidal
ast-1	T08H4.3	Vidal
ceh-43	C28A5.4	Vidal
jun-1	T24H10.7	Vidal
fos-1	F29G9.4	Vidal
amx-2	B0019.1	Vidal

Table S3. ANOVA statistics. Related to Figures 2, 3, 4, and 5.

Figure panel	F-statistic	P-value
**2C and S3A**	F (7, 2852) = 12.51	P<0.0001
**2D and S3B**	F (7, 4364) = 320.7	P<0.0001
**2E and S3C**	F (7, 5218) = 196.9	P<0.0001
**2F and S3D**	F (7, 4657) = 72.38	P<0.0001
**2G and S3E**	F (7, 3547) = 20.65	P<0.0001
**3A**	F (5, 2600) = 147.8	P<0.0001
**3B**	F (3, 563) = 29.36	P<0.0001
**4B**	F (11, 1776) = 22.40	P<0.0001
**4G**	(2-way ANOVA, column factor)<br>F (1, 8738) = 51.20	P<0.0001
**5A**	F (5, 36) = 5.299	P=0.0009
**5B**	F (6, 21) = 7.764	P=0.0002
**5C**	F (5, 17) = 14.46	P<0.0001
**5D**	F (5, 17) = 98.95	P<0.0001
**5F**	F (2, 3781) = 47.80	P<0.0001
